# Structure-activity mapping of ARHGAP36 reveals regulatory roles for its GAP homology and C-terminal domains

**DOI:** 10.1371/journal.pone.0251684

**Published:** 2021-05-17

**Authors:** Patricia R. Nano, Taylor K. Johnson, Takamasa Kudo, Nancie A. Mooney, Jun Ni, Janos Demeter, Peter K. Jackson, James K. Chen

**Affiliations:** 1 Department of Chemical and Systems Biology, Stanford University School of Medicine, Stanford, California, United States of America; 2 Department of Microbiology and Immunology, Stanford University School of Medicine, Stanford, California, United States of America; 3 Department of Developmental Biology, Stanford University School of Medicine, Stanford, California, United States of America; 4 Department of Chemistry, Stanford University, Stanford, California, United States of America; National Cancer Institute, UNITED STATES

## Abstract

ARHGAP36 is an atypical Rho GTPase-activating protein (GAP) family member that drives both spinal cord development and tumorigenesis, acting in part through an N-terminal motif that suppresses protein kinase A and activates Gli transcription factors. ARHGAP36 also contains isoform-specific N-terminal sequences, a central GAP-like module, and a unique C-terminal domain, and the functions of these regions remain unknown. Here we have mapped the ARHGAP36 structure-activity landscape using a deep sequencing-based mutagenesis screen and truncation mutant analyses. Using this approach, we have discovered several residues in the GAP homology domain that are essential for Gli activation and a role for the C-terminal domain in counteracting an N-terminal autoinhibitory motif that is present in certain ARHGAP36 isoforms. In addition, each of these sites modulates ARHGAP36 recruitment to the plasma membrane or primary cilium. Through comparative proteomics, we also have identified proteins that preferentially interact with active ARHGAP36, and we demonstrate that one binding partner, prolyl oligopeptidase-like protein, is a novel ARHGAP36 antagonist. Our work reveals multiple modes of ARHGAP36 regulation and establishes an experimental framework that can be applied towards other signaling proteins.

## Introduction

ARHGAP36 is a multidomain signaling protein with emerging roles in neural development and cancer. This atypical member of the Rho GAP family is expressed in the brain, spinal cord, and endocrine tissues [1–3], and ARHGAP36 deficiency leads to loss of lateral motor column neurons in mouse models [2]. ARHGAP36-dependent spinal cord patterning is likely mediated by Gli transcription factor activation, as ectopic ARHGAP36 expression in the neural tube induces Hedgehog (Hh) target gene expression and ventral cell fates [2]. However, ARHGAP36 does not activate the canonical Hh signaling pathway. While Hh morphogens act through the transmembrane receptors Patched1 (PTCH1) and Smoothened (SMO) to regulate Gli function [4–6], ARHGAP36 induces Gli activation in a SMO-independent manner [7]. This non-canonical mechanism of action likely stems from the ability of ARHGAP36 to promote protein kinase A (PKA) degradation [8], thereby preventing the phosphorylation-dependent proteolysis of GLI2 and GLI3 and enabling these transcription factors to activate *Gli1* and other target genes [9–12].

Consistent with the oncogenic potential of Gli proteins [13, 14], ARHGAP36 dysregulation has been associated with tumorigenesis. *Arhgap36* overexpression in murine cerebellar granule neuron precursors, the cells of origin for certain medulloblastoma subtypes [15], induces Hh ligand-independent Gli activation and proliferation [1, 7]. *Arhgap36* transcription has also been found to correlate with SMO inhibitor resistance in Hh pathway-dependent murine medulloblastomas [7, 16]. ARHGAP36 may promote tumor growth through multiple pathways, as *Arhgap36* has been identified as an oncogenic driver of both Hh pathway-dependent and independent medulloblastomas in mice [1]. Moreover, elevated *ARHGAP36* expression has been observed in Hh pathway-independent subtypes of human medulloblastoma, neuroblastoma, and endocrine cancers [1, 7, 8, 17–19].

**Fig 1:**
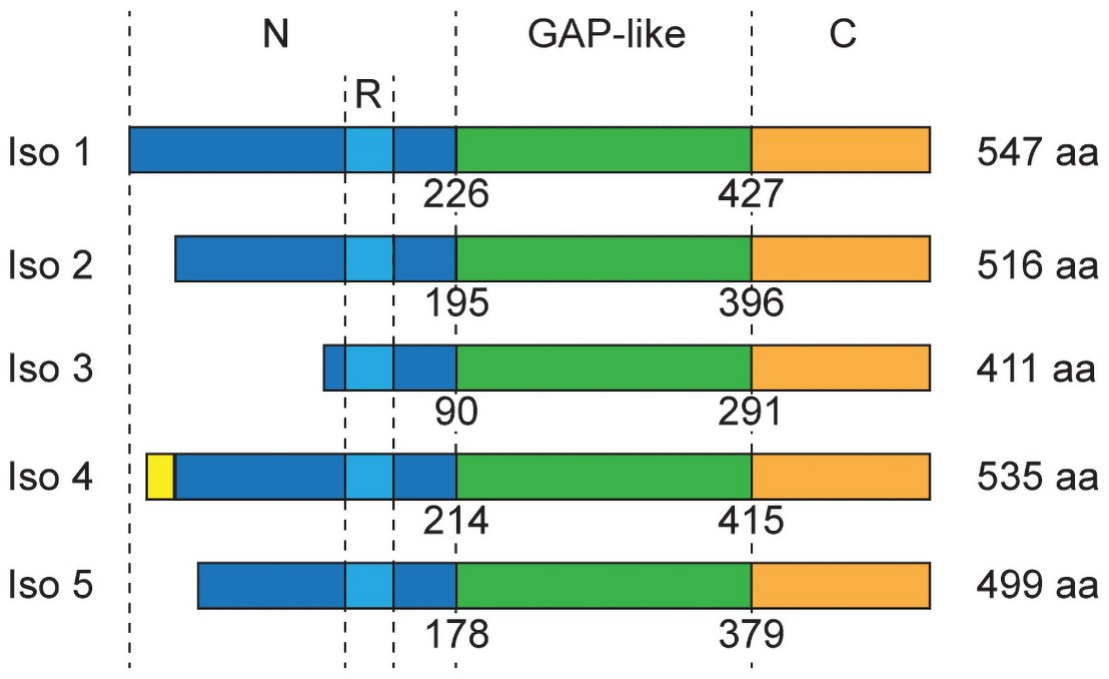
Domain architecture of the five human ARHGAP36 isoforms. The N-terminal domain (N) is shown in dark blue, arginine-rich motif (R) in light blue, GAP-like domain in green, and C-terminal domain (C) in orange. The yellow region indicates an amino acid sequence unique to isoform 4. Residue numbers demarcate the beginning of the GAP-like and C-terminal domains based on the amino acid sequence of each isoform.

While roles for ARHGAP36 in ontogeny and oncogenesis have become increasingly clear, the mechanisms that regulate and transduce ARHGAP36 functions are not well understood. These processes are likely modulated by specific structures within the ARHGAP36 protein, which consists of unique N- and C-terminal domains and a central region that is homologous to Rho GAPs. Human ARHGAP36 is expressed as five splice variants with varying N-terminal sequences (Fig 1). The longest variant (isoform 1) is exclusively expressed in the fetal cerebellum [1], and shorter forms are predominant in subtypes of medulloblastoma (isoforms 2, 3, or 5) and neuroblastoma (isoform 3) [1, 7, 8]. Direct comparisons of the ARHGAP36 isoforms have suggested regulatory roles for the N-terminal domain in ARHGAP36 activity and localization. When overexpressed in cultured cells, isoform 1 adopts a perinuclear distribution and does not affect Gli function, while the other four variants localize to the plasma membrane and can activate Gli transcription factors [7]. Isoform 3 also accumulates within the primary cilium [7], a signaling center that is required for Gli regulation by both ARHGAP36 and the canonical Hh pathway [20–22].

More recently, cell-based studies have demonstrated that an N-terminal arginine-rich motif conserved in all human ARHGAP36 isoforms can inhibit PKA activity by binding directly to the catalytic subunits of this kinase (PRKACA and PRKACB; henceforth referred to as PKA_cat_) [8]. In the context of isoform 2, this motif also mediates PKA_cat_ degradation through a ubiquitin-dependent lysosomal pathway [8]. Furthermore, a 77-amino-acid N-terminal fragment of isoform 2 that includes this arginine-rich region (N2_114-194_) has been shown to be necessary and sufficient for cellular depletion of PKA_cat_ and subsequent expression of Hh target genes such as *Gli1* [8].

Functions of the ARHGAP36 GAP-like and C-terminal domains have not yet been elucidated. Rho GAP family members typically attenuate the activity of Rho GTPases by stimulating GTP hydrolysis [23]. However, the GAP-like region in ARHGAP36 lacks the “arginine finger” motif conserved in catalytically active homologs [7, 24], and ARHGAP36 has no effect on the activities of Rac1, Cdc42, and Rho A [25]. In addition, ARHGAP36 residues that are structurally equivalent to those previously associated with GTP hydrolysis are not required for ARHGAP36-mediated Gli activation [7]. Non-catalytic mechanisms have been reported for several Rho GAP family members [26–28], and it is possible that the GAP homology domain in ARHGAP36 similarly interacts with Rho GTPases or other signaling proteins in a stoichiometric manner. How the C-terminal domain might contribute to ARHGAP36 function is particularly enigmatic since it lacks sequence homology with other proteins.

Deciphering the mechanisms that regulate ARHGAP36 activity requires a more comprehensive map of the ARHGAP36 structure-activity landscape. Here we describe our systematic characterization of this signaling protein using deep-sequencing-based mutagenesis screening, truncation mutant analyses, and comparative proteomics. Our multimodal approach establishes regulatory roles for the GAP-like and C-terminal domains in ARHGAP36 activity, localization, and protein binding. In particular, we have identified several GAP-like domain point mutations distal to the putative Rho GTPase-binding site that abrogate Gli activation. We also demonstrate that the C-terminal domain can counteract an autoinhibitory N-terminal motif that is found in certain ARHGAP36 isoforms. These regulatory mechanisms likely involve changes in ARHGAP36 trafficking, as the autoinhibitory sequence, GAP-like domain, and C-terminal region each modulate ARHGAP36 localization in distinct ways. Finally, we have leveraged our inactive GAP-like domain mutants in a comparative proteomic analysis to discover factors that bind specifically to the active form of ARHGAP36. Among these interactors is the prolyl oligopeptidase-like protein (PREPL), which we demonstrate to be an antagonist of ARHGAP36 expression.

Taken together, this work extends our understanding of ARHGAP36 function beyond the N-terminus and reveals the importance of the GAP-like and C-terminal domains in ARHGAP36 activity and localization. In combination with alternative ARHGAP36 splicing and tissue-specific factors, these regulatory modes could allow ARHGAP36 to alter Gli activity and other PKA_cat_-regulated processes in a context-specific manner. Our studies provide a resource of mutants and activity-dependent interactors that can advance further interrogation of these mechanisms, as well as a multimodal approach that can applied to other signaling proteins.

## Results

### Mutations in the N-terminal and GAP homology domain inhibit ARHGAP36 function

**Fig 2:**
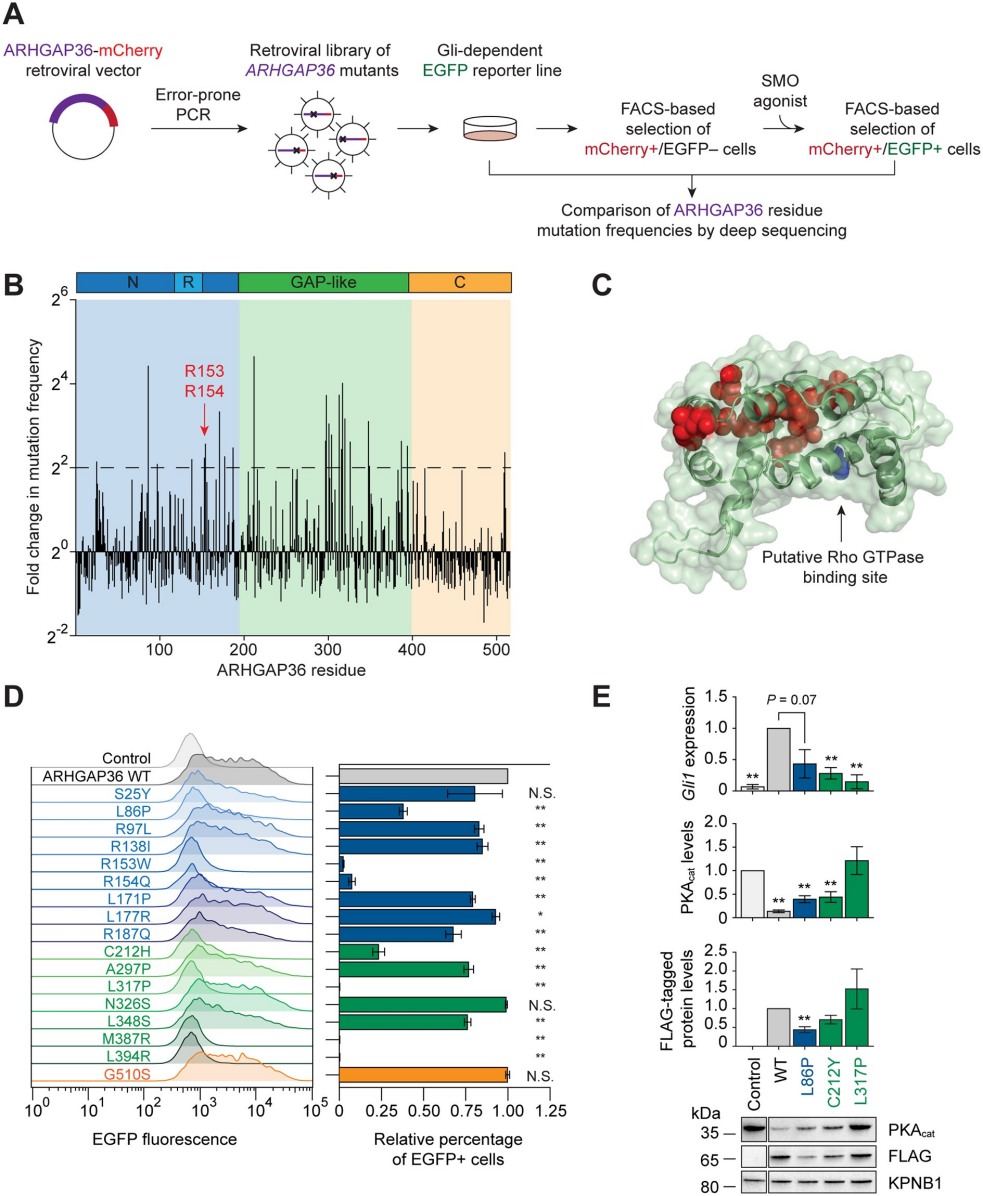
Identification of essential residues within the N-terminal and GAP-like domains. (A) Schematic representation of the high-throughput mutagenesis screen used to identify individual residues that contribute to ARHGAP36 function. (B) Histogram depicting the fold change in mutation frequency between pre- and post-selected populations for each ARHGAP36 residue. (C) Homology model of the GAP-like domain based on the β2-chimaerin crystal structure (PDB ID: 1xa6). Residues with > 4-fold change in mutation frequency are shown in red, and the site that is structurally equivalent to the arginine finger (Thr227) is shown in blue. (D) Activities of selected ARHGAP36-mCherry variants in SHH-EGFP cells, as assessed by flow cytometry. mCherry fluorescence intensities were used to gate cells with comparable levels of ARHGAP36 expression, and the distributions of EGFP fluorescence (left) and relative percentage of EGFP+ cells (right) are shown for each ARHGAP36 construct. Data are the average fold change in percentage of EGFP+ cells relative to that of cells expressing wild-type ARHGAP36 for three biological replicates ± s.e.m. Single and double asterisks indicate *P* < 0.05 and *P* < 0.01, respectively, and N.S. indicates *P* ≥ 0.05. (E) *Gli1* mRNA and PKA_cat_ protein levels in NIH-3T3 cells retrovirally transduced with the indicated FLAG-tagged ARHGAP36 constructs. Uninfected cells were used as controls. Data are the average fold change relative to cells expressing wild-type ARHGAP36 (*Gli1* mRNA or FLAG-tagged protein levels) or to untreated cells (PKA_cat_ levels) for three biological replicates ± s.e.m. Single and double asterisks indicate *P* < 0.05 and *P* < 0.01, respectively. Representative western blots for each condition are shown, with the importin β1 subunit (KPNB1) used as a loading control (lanes from the same blot image have been cropped and re-ordered for clarity).

To identify functional elements within the ARHGAP36 structure, we developed a high-throughput mutagenesis screen for ARHGAP36 residues that are essential for Gli activation (Fig 2A). We applied error-prone PCR on isoform 2, the human variant previously used to study ARHGAP36-dependent PKA_cat_ degradation and Gli activation [8], to create a collection of 100,000 single point mutants fused to mCherry at their C-terminal ends (27% of all library constructs and 30-fold theoretical coverage of the 3,370 possible variants that can arise from single-nucleotide changes to the *ARHGAP36* isoform 2 coding sequence). We then retrovirally transduced the library into NIH-3T3 fibroblasts expressing a Gli-dependent green-fluorescent reporter (SHH-EGFP cells) [29], using a multiplicity of infection (MOI) of 0.3 to maximize the number of cells with single integration events. Cells expressing full-length ARHGAP36 proteins were isolated by fluorescence-activated cell sorting (FACS) according to their mCherry fluorescence. The ARHGAP36 mutant library was then screened under two selection conditions. The cells were first cultured under Hh signaling-competent conditions to allow active ARHGAP36 mutants to induce Gli-dependent EGFP expression, and the population was enriched for inactive ARHGAP36 variants by mCherry+/EGFP–sorting. To ensure that these ARHGAP36-expressing cells still harbored a functional EGFP reporter, they were subsequently cultured with the SMO agonist SAG [30, 31]. mCherry+/EGFP+ cells were obtained by FACS, yielding a population of cells expressing putative, non-functional ARHGAP36-mCherry variants.

To identify inactivating point mutations, we used genomic PCR and deep sequencing to compare the mutation frequency of each amino acid position in the pre- and post-selection populations. This analysis revealed several residues that could be required for Gli activation, including two residues (R153 and R154) in the N-terminal arginine-rich motif that have been previously shown to be required for PKA_cat_ inhibition (Fig 2B, S1 Dataset) [8]. The majority of these putative essential residues were located in the GAP homology region, and structure homology modeling of this domain predicted that these amino acids cluster at a site that is distal to the predicted Rho GTPase-binding pocket (Fig 2C).

We next validated a subset of these ARHGAP36 point mutants using flow cytometry-based assays. These studies surveyed all N- and C-terminal residues that were mutated > 4-fold more frequently in the inactive mutant pool and a representative sample of GAP-like domain residues that passed this criterion. The tested variants incorporated the mutation most enriched in the inactive pool for the selected sites, and each was individually transduced into cells at an MOI of 0.3. Point mutations in the N-terminal domain (L86P, R153W, R154Q) and GAP-like region (C212H, L317P, M387R, and L394R) decreased ARHGAP36 activity in SHH-EGFP reporter cells to the greatest extent (Fig 2D). We also investigated how individual mutations might affect ARHGAP36 functions in NIH-3T3 cells, focusing on the three residues with the greatest fold change in pre- and post-selection mutation frequencies. These experiments assessed the L86P and L317P mutants described above and another C212 variant identified in our screen, C212Y (S1 Fig). The L86P mutant was less able to induce PKA_cat_ degradation and *Gli1* expression, but this reduced activity coincided with a corresponding decrease in ARHGAP36 levels (Fig 2E). In comparison, the C212Y and L317P variants had diminished specific activities, with the latter mutation causing almost complete loss of ARHGAP36 action on PKA_cat_ and *Gli1* (Fig 2E). Collectively, these results reveal an important role for the GAP homology domain in ARHGAP36 signaling.

### The C-terminal domain counteracts an autoinhibitory N-terminal sequence

**Fig 3:**
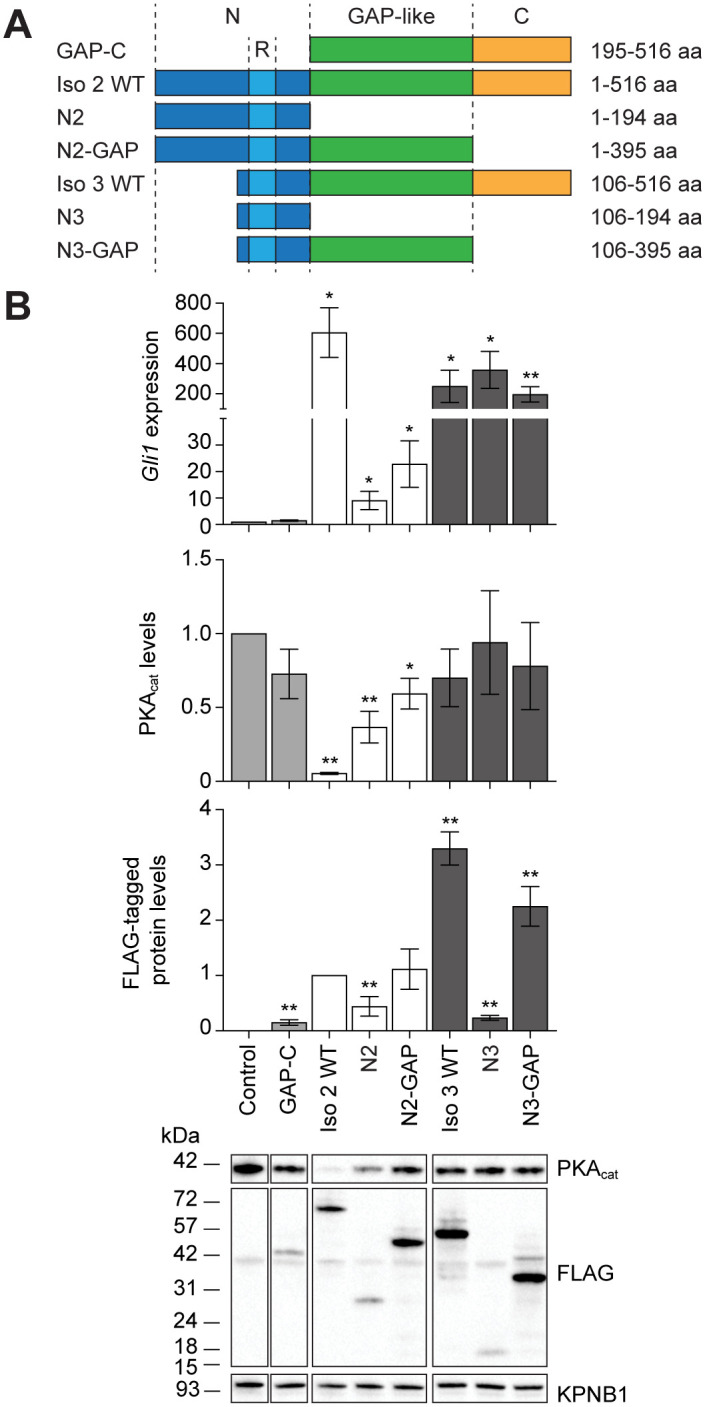
The C-terminal domain counteracts an autoinhibitory N-terminal sequence. (A) Schematic representation of ARHGAP36 isoform 2 or 3 truncation mutants. Residue numbers are based on the amino acid sequence of isoform 2. (B) *Gli1* mRNA and PKA_cat_ protein levels in NIH-3T3 cell retrovirally transduced with the indicated FLAG-tagged ARHGAP36 truncation mutants. Data are the average fold change relative to uninfected cells (*Gli1* mRNA or PKA_cat_ protein levels) or to cells expressing ARHGAP36 isoform 2 (FLAG-tagged protein levels) for three biological replicates ± s.e.m. Single and double asterisks indicate *P* < 0.05 and *P* < 0.01, respectively. Representative western blots for each condition are shown, with the importin β1 subunit (KPNB1) used as a loading control (lanes from the same blot image have been cropped and re-ordered for clarity).

The prevalence of inactivating mutations within the GAP homology region is surprising given the sufficiency of an N-terminal polypeptide for ARHGAP36 function [8]. We therefore used truncation mutant analyses to uncover functional relationships between N-terminal sequences and other regions in the ARHGAP36 protein, as well as possible differences between ARHGAP36 isoforms (Fig 3A). These studies focused on two representative ARHGAP36 splice variants that can activate Gli proteins: isoform 2, which was used in our mutagenesis screen, and isoform 3, a predominant species detected in medulloblastomas and neuroblastomas [1, 7, 8].

By retrovirally expressing these constructs in NIH-3T3 cells, we observed that the N-terminal domains of ARHGAP36 isoform 2 (N2) and isoform 3 (N3) can induce *Gli1* expression (Fig 3B). However, the two N-terminal variants exhibited significant functional differences. N2 was much less effective at activating *Gli1* expression than N3, even though the two truncation mutants were expressed at comparable levels (Fig 3B). Since N2 and N3 differ only in their N-terminal-most sequences, these findings demonstrate that residues 1–105 in N2 (N2_1-105_) strongly attenuate ARHGAP36 action on Gli proteins, presumably by affecting its ability to target the PKA_cat_ pool that regulates these transcription factors.

These studies also revealed that both N2 and N2-GAP are significantly less active than full-length isoform 2 (Fig 3B), indicating that the C-terminal domain is essential for the maximal activity of this splice variant. N2 and N2-GAP appear to have comparable potencies, as the expression level and Gli-activating potency of N2 were both moderately lower than those of N2-GAP. In contrast, N3-GAP could induce *Gli1* transcription to a similar extent as full-length isoform 3, and the N3 construct appeared to have much greater activity despite the low expression level of this variant (Fig 3B). These observations indicate that the C-terminal domain can counteract the repressive activity of N2_1-105_ and suggest that the GAP-like domain can also negatively regulate N-terminal domain functions.

In addition to the N2_1-105_ motif, our analyses revealed another unexpected difference between isoforms 2 and 3. Although both full-length proteins could effectively induce *Gli1* expression, isoform 3 did not deplete cellular PKA_cat_ levels to a discernable extent (Fig 3B). Previous studies have shown that isoform 3 can accumulate in the primary cilium [7], and we surmised that this splice variant might selectively suppress a subcellular pool of PKA_cat_ that regulates Gli proteins. We therefore used immunofluorescence microscopy to compare the effects of isoforms 2 and 3 on the subcellular distribution of PKA_cat_, finding that isoform 2 globally depleted PKA_cat_ in NIH-3T3 cells, whereas isoform 3 preferentially reduced PKA_cat_ levels in the Golgi (S2 Fig).

### The GAP-like and C-terminal domains regulate ARHGAP36 localization

**Fig 4:**
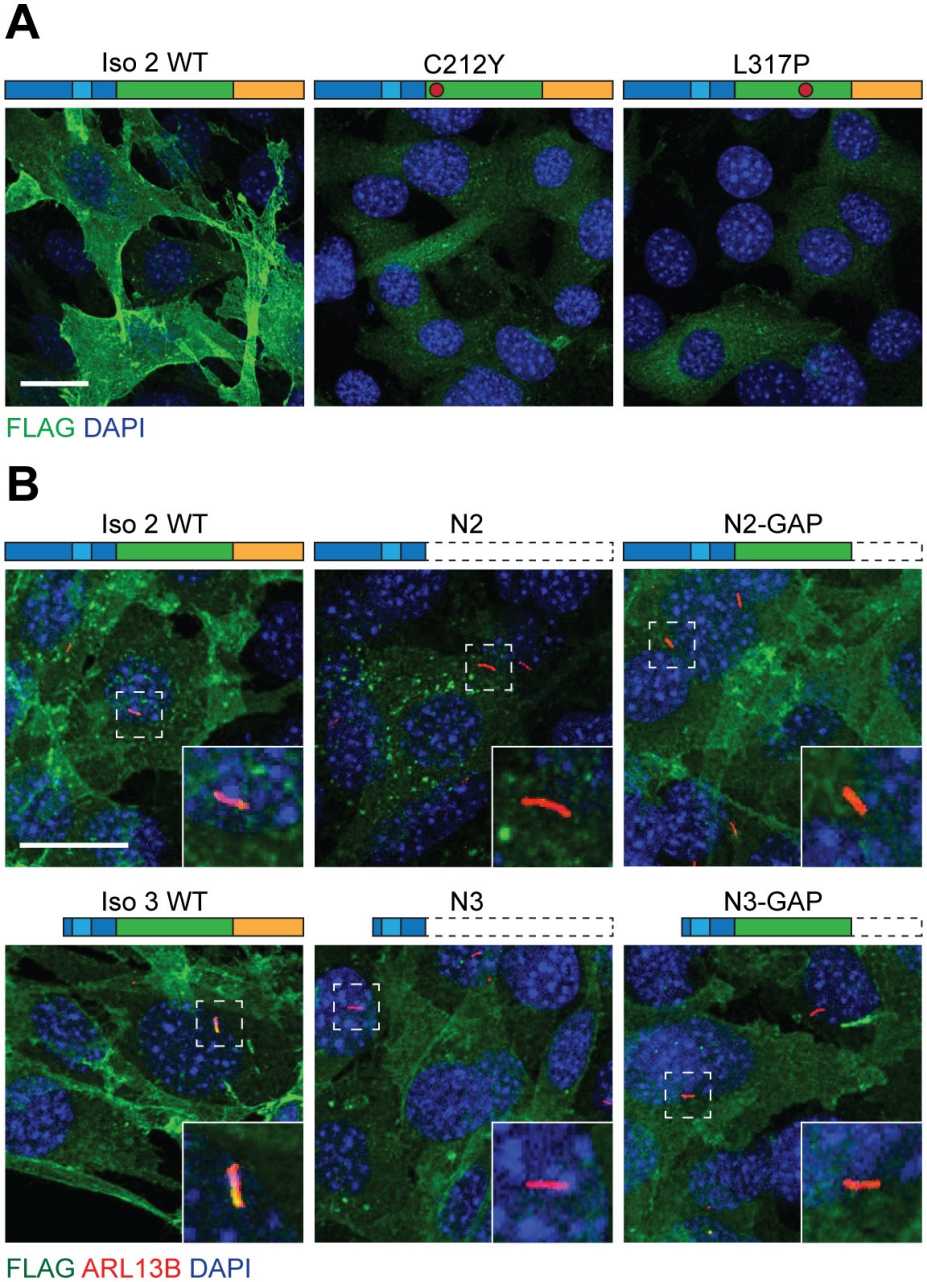
The GAP-like and C-terminal domains regulate ARHGAP36 localization. Subcellular distributions of the indicated FLAG-tagged ARHGAP36 point mutants (A) and truncation mutants (B) in NIH-3T3 cells. Representative maximum-intensity Z-stack projections are shown with immunofluorescent staining for FLAG and DAPI (nucleus) (A) or FLAG, DAPI, and ARL13B (primary cilium) (B). Insets highlight ciliated regions in the dashed boxes. Scale bar: 20 μm. Images were processed to establish comparable maximum pixel intensities in order to highlight differences in localization.

We next sought to examine how the GAP-like and C-terminal domains might regulate ARHGAP36 activity. Since N-terminal sequences can alter ARHGAP36 function and trafficking, we investigated whether the GAP homology and C-terminal regions could also modulate ARHGAP36 localization in NIH-3T3 cells. We first surveyed the subcellular distributions of the C212Y and L317P mutants of ARHGAP36 isoform 2, observing that both GAP-like domain mutations rendered ARHGAP36 cytosolic (Fig 4A). We extended these studies to other inactivating mutations identified in our high-throughput screen, finding that other GAP homology mutations (C212H, M387R, and L394R) but not N-terminal mutations (R153W, R154Q) suppressed ARHGAP36 recruitment to the plasma membrane (S3 Fig). The GAP-like domain point mutations similarly abrogated the function and localization of isoform 3 (S4 Fig).

By comparing the subcellular distributions of our ARHGAP36 truncation mutants in NIH-3T3 cells, we also discovered the ability of other ARHGAP36 structures to regulate protein trafficking. For example, N2 predominantly localized to internal punctate structures, while N3 and N2-GAP accumulated at the plasma membrane (Fig 4B). These observations suggest a role for the N2_1-105_ motif in restricting movement of ARHGAP36 to the plasma membrane and the ability of the GAP homology domain to counteract this activity. Full-length isoform 3, N3, and N3-GAP also exhibited differing localizations. All three constructs trafficked to the plasma membrane, but only the full-length protein accumulated within the primary cilium, uncovering a role for the C-terminal domain in promoting ARHGAP36 transport to this signaling center (Fig 4B).

The inhibitory effects of GAP-like domain point mutations on both ARHGAP36 Gli-activating function and plasma membrane localization contrast the sufficiency of N2 and N3 for these activities. Taken together, these results suggest that in addition to its ability to counteract the localization effects of N2_1-105_, the GAP-like domain may repress the conserved N-terminal sequences that promote Gli activation and plasma membrane targeting. To test for direct interactions between these two domains, we co-transfected HEK-293 cells with constructs encoding FLAG-tagged N3 and the GAP homology region fused to a LAP tag (S-peptide-PreScission protease site-EGFP) [32–37] and pulled down the LAP-tagged GAP-like domain from the resulting cell lysates. Western blot analyses detected the co-immunoprecipitation of N3-FLAG and the LAP-tagged GAP construct, suggesting that the GAP domain could regulate N-terminal functions through intra- or intermolecular interactions (S5 Fig).

### GAP-like domain mutations alter the ARHGAP36 interactome

**Fig 5:**
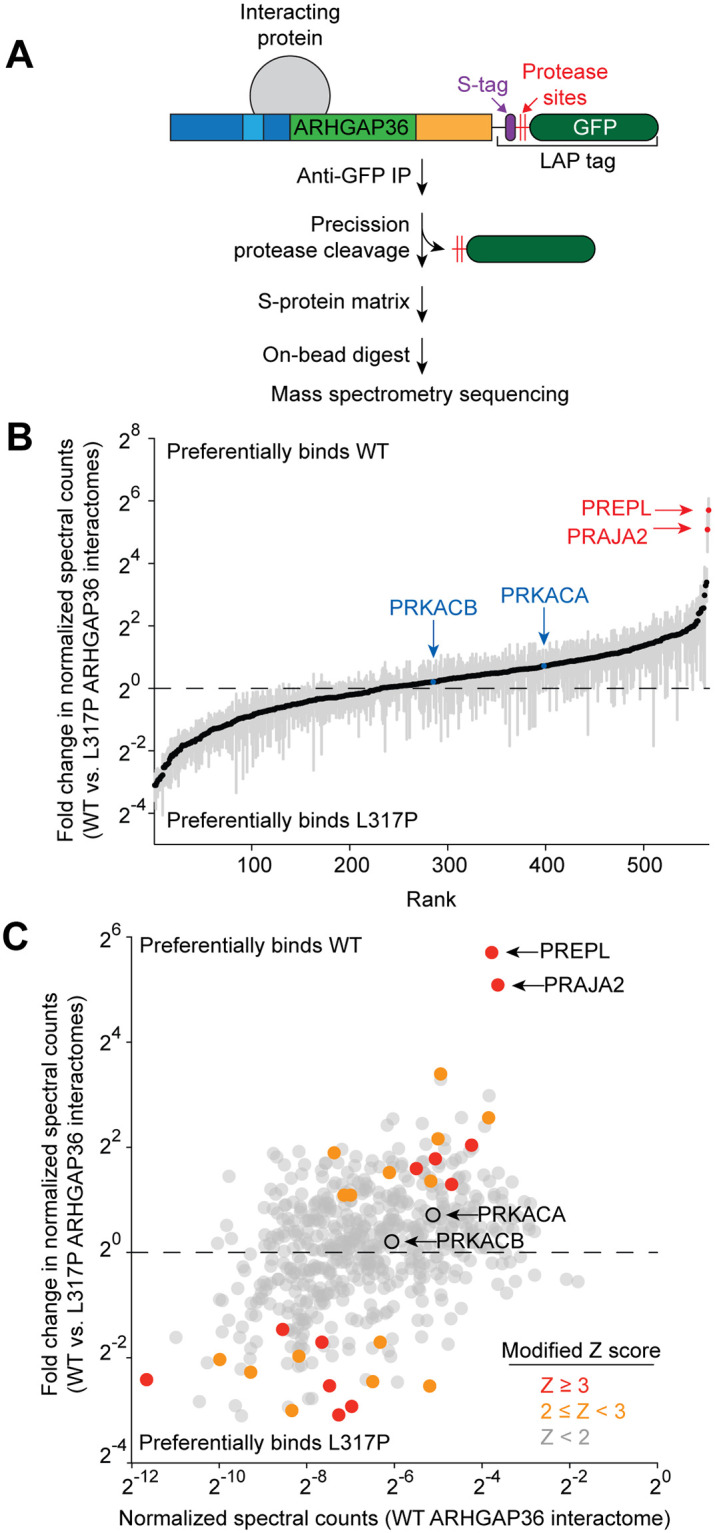
PREPL and PRAJA2 interact with ARHGAP36 in a GAP-like domain-dependent manner. (A) Schematic representation of the tandem affinity purification workflow for identifying ARHGAP36-binding proteins. (B) ARHGAP36 interactors ranked in order of their relative binding to the wild-type versus L317P proteins. Fold changes in normalized spectral counts represent the average values for three biological replicates ± s.e.m., shown as grey bars. (C) Scatter plot of ARHGAP36 isoform 2-binding proteins according to their normalized spectral counts in the wild-type and L317P interactomes. Each data point represents the average value for three biological replicates, and a modified Z-score was calculated for each fold change in abundance (see Methods).

As reported for several non-catalytic Rho GAP family members [26–28], the GAP-like domain may alter ARHGAP36 function by mediating protein-protein interactions. Cellular factors may also modulate the dual functions of the GAP-like domain. We systematically investigated these hypotheses by comparing the interactomes of wild-type and L317P ARHGAP36 isoform 2. NIH-3T3 cells were retrovirally transduced with vectors encoding each ARHGAP36 construct fused to a C-terminal LAP tag [32–37] (Fig 5A). To avoid total PKA_cat_ depletion by wild-type ARHGAP36 in these studies, we also limited the cells to a 4-hour incubation in retroviral medium and a subsequent 20-hour growth phase. The fibroblasts were then lysed, and each ARHGAP36 construct and its interacting proteins were isolated by tandem affinity purification and proteolytically digested as previously described [32–35, 37]. The resulting peptides were sequenced and quantified using tandem mass spectrometry, and spectral counts were normalized to account for variabilities in protein size, LAP tag purification efficiency, and ARHGAP36 expression.

Our approach identified 566 putative interactors for wild-type and/or L317P ARHGAP36 that were observed across three biological replicates (Fig 5B, S2 Dataset). PKA_cat_ subunits were the only canonical Hh pathway regulators detected in these pulldown experiments, and they interacted with wild-type and L317P ARHGAP36 to similar extents. Interestingly, factors that preferentially bound to active ARHGAP36 were not necessarily the most abundant proteins in the wild-type interactome (Fig 5C, S2 Dataset). Our dataset therefore facilitates the identification of binding proteins that specifically modulate or transduce the functions of active ARHGAP36.

Among these interactors, prolyl oligopeptidase-like protein (PREPL) and the E3 ubiquitin ligase PRAJA2 had binding selectivities with the greatest magnitude (52- and 35-fold, respectively) and high statistical significance (modified Z-scores of 4.50 and 4.26, respectively). PRAJA2 has been shown to increase PKA activity by promoting the ubiquitination and degradation of regulatory PKA (PKA_reg_) subunits, a function that is enhanced by PKA_cat_ phosphorylation as part of a positive-feedback mechanism [38–40]. PREPL has β-propeller and serine hydrolase-like domains [41], and genetic loss of PREPL function has been linked to hypotonia in humans and mouse models [42, 43]. However, the physiological substrates and molecular functions of this putative enzyme are unknown. Both PREPL and PRAJA2 have been classified as putative ARHGAP36-binding partners in large-scale interactome studies [25, 44], but their functional significance relative to the other candidates in those lists has not yet been determined.

### PREPL suppresses ARHGAP36-mediated Gli activation

**Fig 6:**
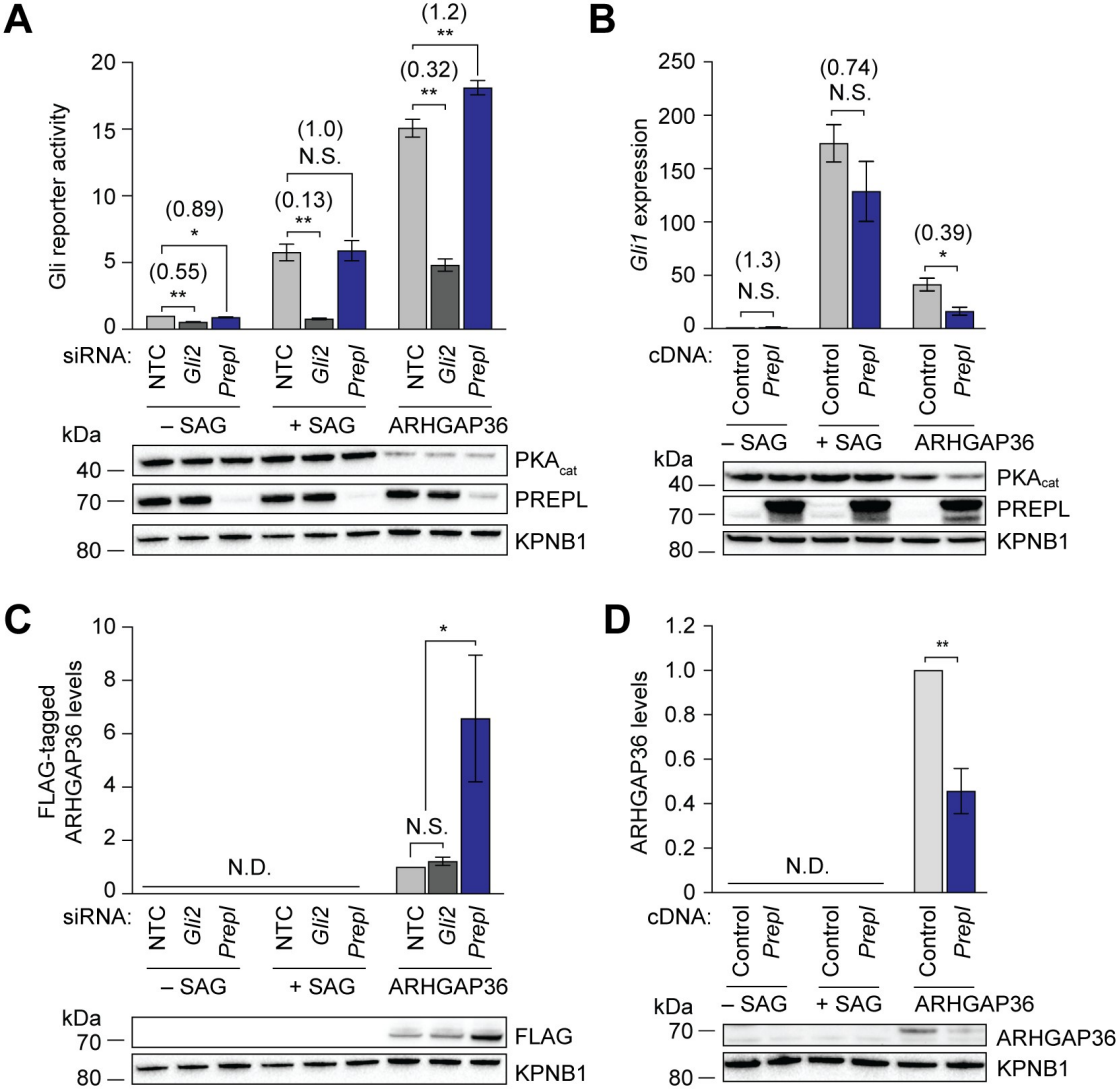
PREPL attenuates ARHGAP36-mediated Gli activation. (A and C) Effects of *Prepl* siRNAs on Gli-dependent luciferase reporter activity (A) and ARHGAP36 protein levels (C) in SHH-LIGHT2 cells stimulated with SAG or retrovirally transduced with FLAG-tagged ARHGAP36 isoform 2. SHH-LIGHT2 cells treated with either a non-targeting control siRNA (NTC) or *Gli2* siRNA were included as controls. Data are the average fold change relative to NTC siRNA-transfected, untransduced cells for six biological replicates ± s.e.m. (A) or ARHGAP36-expressing cells for five biological replicates ± s.e.m. (C). (B and D) Effects of exogenous *Prepl* expression on *Gli1* transcription (B) and ARHGAP36 protein levels (D) in NIH-3T3 cells stimulated with SAG or transduced with FLAG-tagged ARHGAP36 isoform 2. Cells transduced with 3xFLAG-ires-mCherry vector were included as negative controls. Data are the average fold change relative to control vector-transduced, unstimulated cells (B) or ARHGAP36-expressing cells (D) for three biological replicates ± s.e.m. Values in parentheses represent the fold change relative to cells transfected with NTC siRNA (A) or transduced with control vector (B) within the same treatment group. Single and double asterisks indicate *P* < 0.05 and *P* < 0.01, respectively, and N.S. indicates P ≥ 0.05. N.D. indicates a lack of detected signal. Representative western blots for each condition are shown, with the importin β1 subunit (KPNB1) used as a loading control.

To determine whether PREPL or PRAJA2 functionally interact with ARHGAP36/Gli signaling, we assessed the effects of knocking down or overexpressing these proteins. Transfection of *Praja2* siRNAs in NIH-3T3 fibroblasts stably transfected with a Gli-dependent firefly luciferase reporter (SHH-LIGHT2 cells) [45] modestly attenuated Gli activity induced by the SMO agonist SAG or ARHGAP36 isoform 2 expression (S6 Fig). Conversely, overexpressing *Praja2* in NIH-3T3 cells had no significant effect on either mechanism of Gli activation as gauged by qRT-PCR (S6 Fig). In comparison, *Prepl* knockdown and overexpression selectively potentiated and inhibited ARHGAP36-mediated Gli activation, respectively (Fig 6A and 6B). To investigate the molecular basis for these functional interactions, we next examined the effects of PREPL on ARHGAP36 expression. PREPL depletion signficantly increased ARHGAP36 protein levels, whereas introducing exogenous *Prepl* had the opposite effect (Fig 6C and 6D). Together, these findings establish PREPL as a suppressor of ARHGAP36 function that acts at least in part by decreasing its protein expression.

In principle, the GAP homology domain in ARHGAP36 could directly interact with PREPL or influence the binding capabilities of other ARHGAP36 regions. To explore the ARHGAP36-PREPL interaction further, we compared the abilities of full-length isoform 2, N2, N2-GAP, and GAP-C to bind this β-propeller-containing protein. We retrovirally transduced NIH-3T3 cells with expression vectors encoding each of these variants with a C-terminal FLAG tag and isolated them from the resulting cell lysates by immunoprecipitation. Western blot analyses failed to detect PREPL in the N2 immunoprecipitates, but the truncation mutants containing the GAP-like domain (N2-GAP and GAP-C) retained the ability to interact with this protein, albeit with lower efficiency than the full-length isoform (S7 Fig). These results indicate that PREPL regulates ARHGAP36 function at least in part through direct interactions with the GAP-like domain.

## Discussion

**Fig 7:**
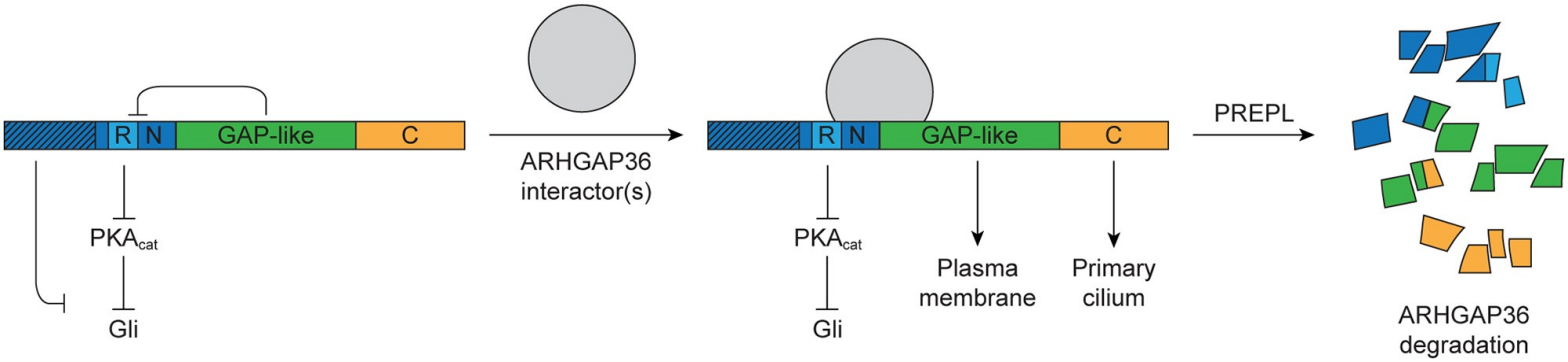
A regulatory model for ARHGAP36 function. Schematic representation of the structural elements that regulate ARHGAP36 localization and activity. N-terminal regulatory regions that vary between ARHGAP36 isoforms are depicted with hash marks.

By systematically exploring the ARHGAP36 structure-activity landscape, we have gained new insights into the molecular mechanisms that regulate this developmental and proto-oncogenic signaling protein. Previous investigations have defined a conserved N-terminal region in ARHGAP36 that is necessary and sufficient for PKA_cat_ inhibition and *Gli1* expression [8], and isoform-specific differences have also implicated other N-terminal sequences in ARHGAP36 trafficking [1, 7]. Our studies expand upon these known mechanisms of ARHGAP36 signaling, establish functional roles for the GAP-like and C-terminal domains, and identify cellular factors that preferentially interact with the active protein (Fig 7).

Among our key findings are regulatory functions of the GAP homology region. Point mutations in this central domain abrogate the ability of ARHGAP36 to deplete cellular PKA_cat_ and activate Gli proteins. The mutations also disrupt ARHGAP36 trafficking, mislocalizing the signaling protein from the plasma membrane to the cytosol. These inhibitory effects might appear to contradict the functional sufficiency of N2 and N3. However, our observations could be explained if the GAP-like domain can repress the conserved N-terminal region that contains the PKA_cat_-inhibiting arginine-rich motif and a plasma membrane-targeting sequence. Consistent with this idea, our truncation mutant analyses demonstrated that the potency of N3 is significantly greater than that of N3-GAP. Our results also provide evidence of direct binding between the N3 and the GAP homology region, indicating that the GAP-like domain may repress these conserved N-terminal sequences through intra- or intermolecular mechanisms. The binding of specific cellular factors to the GAP-like domain could then modulate this interaction and other ARHGAP36 activities (see Fig 7). Putative ARHGAP36-binding proteins are not necessarily limited to Rho GTPases, as the inactivating point mutations identified in our high-throughput screen are distal to the putative Rho GTPase binding site [7, 24]. In addition, other Rho GAPs lacking the catalytic arginine finger have been found to mediate non-catalytic protein-protein interactions [26–28].

Our experimental results also establish the N- and C-terminal domains as important regulators of ARHGAP36 function. Isoform 2 contains an autoinhibitory motif that is counteracted by the C-terminal region, which is necessary for the maximal activity of this ARHGAP36 splice variant. Conversely, isoform 3 does not require its C-terminal domain to achieve high levels of *Gli1* expression, and other ARHGAP36 variants that lack the N2_1-105_ sequence could function in a similar manner. We note that the autoinhibitory motif is absent from N2_118-194_, the N-terminal fragment that is sufficient for PKA_cat_ degradation and Gli activation [8]. In addition, mutations in the *Arhgap36* locus that result in N-terminally-truncated protein products can drive medulloblastoma formation in mice, while normal fetal cerebellum only expresses ARHGAP36 isoform 1, which contains the longest N-terminal domain of all ARHGAP36 splice variants [1]. Most recently, an ARHGAP36 L94F variant was identified by whole-exome sequencing of spina bifida patients as a potential contributor to this neural tube defect [46], which can be caused by dysregulated Hh pathway activation [47]. Coupled with our results, these findings raise the possibility that differences in the N-terminal regions of ARHGAP36 variants may alter the presence of autoinhibitory sequences and result in isoform- or allele-specific functions. Our studies further demonstrate that the C-terminal domain can promote ARHGAP36 trafficking to the primary cilium. In the context of isoform 3, this function leads to the steady-state ciliary accumulation of ARHGAP36 and the preferential depletion of Golgi-localized PKA_cat_, likely due to vesicular transport between these subcellular compartments [48]. N2_1-105_ may attenuate the ability of the C-terminal domain to induce ciliary accumulation, enabling ARHGAP36 variants such as isoform 2 to more readily access and deplete other cellular PKA_cat_ pools.

The regulatory activities of the GAP homology and C-terminal domains could involve the recruitment of other cellular factors. To facilitate the discovery of proteins that modulate or transduce ARHGAP36-dependent Gli activation, we have compared the interactomes of wild-type ARHGAP36 and an inactive GAP-like domain mutant. This approach not only identifies ARHGAP36 interactors but also enables the prioritization of those that bind selectively to the active protein. Although previous studies have shown that ARHGAP36 can co-immunoprecipitate with PTCH1 [49] and SUFU [7] when they are overexpressed in cells, we did not observe analogous interactions with the endogenous Hh signaling proteins. Rho GTPases were also notably absent from the ARHGAP36 pulldowns, suggesting that ARHGAP36 interacts transiently with these signaling proteins or prefers other binding partners. In contrast, PKA_cat_ associated with both wild-type and L317P ARHGAP36 with comparable efficacies, indicating that the GAP-like domain mutation abrogates PKA_cat_ degradation without compromising its binding. Among the ARHGAP36 interactors discovered in our studies, PREPL and PRAJA2 emerged as the two most sensitive to the L317P mutation. In principle, the GAP-like domain could bind directly to PREPL or PRAJA2, allosterically modulate their interactions with other ARHGAP36 domains, regulate the subcellular co-localization of ARHGAP36 with these factors, or act through a combination of these mechanisms.

Both PREPL or PRAJA2 were candidates in previous ARHGAP36 interactome datasets [25, 44], but the functional relevance of these factors and other ARHGAP36-binding proteins has yet to be determined. By comparing the wild-type and mutant ARHGAP36 interactomes, our proteomic analyses provide a functional context for these binding proteins, implicating PREPL and PRAJA2 in ARHGAP36 signaling. Our functional studies indicate that PRAJA2 does not play a major role in ARHGAP36-mediated Gli activation. This E3 ligase has well-established functions in PKA_reg_ degradation and PKA_cat_ activation [38–40], and we anticipate that ARHGAP36-PRAJA2 interactions might modulate the ability of ARHGAP36 to control other PKA-regulated processes. For example, PRAJA2 might underlie the association of ARHGAP36 with Hh pathway-independent medulloblastoma subtypes, neuroblastoma, and/or endocrine cancers [1, 7, 8, 17–19]. In comparison, our findings establish PREPL as an antagonist of ARHGAP36-mediated Gli activation. PREPL action on Gli proteins appears to be specific for this non-canonical pathway, as PREPL does not modulate SAG-induced Gli activation. Nor was PREPL identified as a Hh pathway regulator in CRISPR knockout screens [50, 51]. Accordingly, we observe that PREPL expression inversely correlates with ARHGAP36 levels, suggesting that this β-propeller protein might promote ARHGAP36 degradation. Since PREPL preferentially interacts with wild-type ARHGAP36 rather than the inactive L317P mutant, we hypothesize that ARHGAP36 activation might coincide with a conformation change that is required for PREPL recruitment. Alternatively, it is possible that wild-type ARHGAP36 traffics through subcellular compartments where binding to PREPL can occur, while the cytosolic L317P mutant is largely excluded from these sites.

The molecular mechanisms by which PREPL suppresses ARHGAP36 expression and function remain to be determined. Our co-immunoprecipitation studies indicate that PREPL can bind directly to the GAP homology domain, which could alter N-terminal domain functions and/or ARHGAP36 localization. There may also be a role for catalytic processes, as PREPL is highly homologous to the prolyl oligopeptidase (PREP) family of serine hydrolases. The hydrolytic targets of PREPL remain unknown [41, 42], and possible substrates may not be limited to peptides. For example, there is evidence that PREP enzymes can modulate phosphoinositides [52, 53], lipids with reported roles in the formation of Golgi-derived vesicles [54, 55] and ciliary trafficking [56, 57]. The physiological context for PREPL-ARHGAP36 signaling also awaits further investigation. While PREPL is expressed in multiple tissues, it is most highly transcribed in the central nervous system [43, 58]. One intriguing possibility is that PREPL regulates ARHGAP36-dependent motor neuron development, which could explain why PREPL deficiency causes hypotonia in humans and mice [42, 43].

Taken together, our findings provide new insights into the mechanisms that underlie non-canonical Gli activation by ARHGAP36 and a general framework for understanding ARHGAP36 function. The regulatory modes revealed by our studies could enable context-specific ARHGAP36 signaling, directed by the expression of specific splice variants and ARHGAP36-binding proteins. We anticipate that these processes not only contribute to Gli-dependent spinal cord development and medulloblastoma progression but also other PKA_cat_-dependent pathways in normal physiology and disease. The ARHGAP36 mutants and interactors identified through our studies will be valuable tools for dissecting these unique functions. Moreover, our coupling of high-throughput mutagenesis screening and comparative proteomics is a versatile approach that could elucidate the structure-activity landscapes of other signaling proteins.

## Materials and methods

### Reagents and cell lines

Antibody sources and working dilutions are listed in S1 Table. SAG was purchased from Tocris. SHH-LIGHT2 [45] and SHH-EGFP cells [29] were described previously, and NIH-3T3 and HEK-293 and HEK-293T cells were purchased from the American Type Culture Collection.

### Expression vectors

The pDONR223 vector was provided by J. Hartley and D. Esposito. The following constructs have been described previously [7, 33, 50]: Gateway cloning destination vectors pBMN-3xFLAG-IRES-mCherry-DEST, pBMN-mCherry-DEST, and pG-LAP7-DEST, Gateway cloning entry vectors pDONR223-ARHGAP36 (isoforms 1–3), and expression vectors pcDNA3.2-ARHGAP36 (isoform 2)-V5, pBMN-3xFLAG-IRES-mCherry, pBMN-mCherry, and pHR-Pgk-Cas9-BFP vector. pCL-ECO retrovirus packaging vector was purchased from Imegenex.

Retroviral expression vectors for FLAG-tagged ARHGAP36 isoforms 2 and 3 were produced by transferring cDNAs from the appropriate pDONR223 entry vectors into pBMN-3xFLAG-IRES-mCherry-DEST in an LR Clonase II (Invitrogen)-mediated recombination reaction. Retroviral expression vectors for mCherry-tagged ARHGAP36 isoforms 1 and 2 were produced in an analogous manner with pBMN-mCherry-DEST. Restriction sites were then inserted upstream (XhoI) and downstream (SacII) of the *ARHGAP36* sequence in the initial pBMN-ARHGAP36 (isoform 1)-mCherry and pBMN-ARHGAP36 (isoform 2)-mCherry products. This was achieved by first amplifying *ARHGAP36* cDNA from the pBMN-derived vectors (S1 Table; primers 1–3) and then inserting the resulting PCR product into BamHI-digested pBMN-mCherry using Gibson assembly (New England Biolabs). The resulting pBMN-ARHGAP36-mCherry vectors with XhoI and SacII restriction sites were subsequently used in all experiments described herein.

Retroviral expression vectors for ARHGAP36 isoform 2 truncation mutants were generated by amplifying the cDNA for each variant from pcDNA3.2-ARHGAP36 (isoform 2)-V5, using primers containing attB adapter sequences (S1 Table; primers 4–8). The PCR products were transferred into pDONR223 in a BP Clonase II (Invitrogen)-mediated recombination reaction, and the *ARHGAP36*-derived cDNAs in these pDONR223 entry vectors were then transferred to pBMN-3xFLAG-IRES-mCherry-DEST using LR Clonase II. The resulting constructs were also used as templates to amplify cDNAs for the analogous FLAG-tagged ARHGAP36 isoform 3 truncation mutants, a GAP-like domain truncation mutant, and the downstream IRES-mCherry sequence (S1 Table; primers 9–14). These PCR products were then inserted into XcmI-digested pBMN-ARHGAP36 (isoform 2)-3xFLAG-IRES-mCherry using Gibson assembly.

Individual ARHGAP36 isoform 2 point mutants, with the exception of L171P, were generated using site-directed mutagenesis with PfuUltra II Fusion polymerase (Agilent) (S1 Table; primers 15–46) and either pBMN-ARHGAP36 (isoform 2)-3xFLAG-IRES-mCherry or the pBMN-ARHGAP36 (isoform 2)-mCherry as template. ARHGAP36 isoform 2 L171P mutant constructs were generated by Gibson assembly using a XhoI- and SacII-digested pBMN-ARHGAP36 (isoform 2)-mCherry plasmid, inserts amplified from pBMN-ARHGAP36 (isoform 2)-mCherry (S1 Table; primers 3 and 47–49), and a double-stranded oligonucleotide encoding the L171P mutation (Integrated DNA Technologies) (S1 Table; entry 50).

Retroviral expression vectors for mCherry-tagged wild-type, C107Y, and L212P ARHGAP36 isoform 3 were generated by amplifying the cDNAs encoding this isoform from the corresponding mutant pBMN-ARHGAP36 (isoform 2)-mCherry plasmids (S1 Table; primers 3 and 51) and amplifying the mCherry tag from pBMN-ARHGAP36 (isoform2)-mCherry plasmid (S1 Table; primers 10 and 52). The resulting amplicons were inserted into XcmI-digested pBMN-ARHGAP36 (isoform 2)-3xFLAG-IRES-mCherry using Gibson assembly.

To generate retroviral expression vectors for LAP-tagged ARHGAP36 constructs, *ARHGAP36* cDNA in the pDONR223-ARHGAP36 (isoform 2) entry vector was transferred to pG-LAP7-DEST using LR Clonase II. The cDNA encoding LAP-tagged ARHGAP36 was amplified from the resulting pG-ARHGAP36 (isoform 2)-LAP7 plasmid (S1 Table; primers 10 and 53) and inserted into XcmI-digested pBMN-ARHGAP36 (isoform 2)-3xFLAG-IRES-mCherry using Gibson assembly. Retroviral constructs encoding LAP-tagged versions of L317P ARHGAP36 isoform 2 or the GAP-like domain truncation mutant were generated using an analogous Gibson assembly with *ARHGAP36* cDNA amplified from pBMN vectors harboring the FLAG-tagged versions of these variants (S1 Table; primers 53–56) and LAP tag cDNA amplified from either the pG or pBMN plasmids encoding ARHGAP36 (isoform 2)-LAP7 (S1 Table; primers 10, 52, and 57). A similar approach was used to create control pBMN-GFP constructs using *GFP* cDNA amplified from pBMN-ARHGAP36 (isoform 2)-LAP7 (S1 Table; primers 10 and 58).

Retroviral *Prepl* and *Praja2* expression vectors were generated using *Prepl* cDNA amplified from NIH-3T3 cDNA (S1 Table; primers 59–60) and murine *Praja2* cDNA purchased from GeneScript. The cDNAs were then amplified (S1 Table; primers 61–64) to attach flanking sequences that enabled Gibson assembly with an IRES sequence amplified from the pBMN-ARHGAP36 (isoform 2)-3xFLAG-IRES-mCherry plasmid (S1 Table; primers 65–67), TagBFP cDNA amplified from pHR-Pgk-Cas9-BFP (S1 Table; primers 68–69), and XcmI-digested pBMN-ARHGAP36 (isoform 2)-3xFLAG-IRES-mCherry.

With the exception of the constructs generated by site-directed mutagenesis described above, all PCR products were generated with Phusion polymerase (New England Biolabs). All plasmids were sequence-verified.

### Retrovirus production

HEK-293T cells were seeded into individual wells of a 6-well plate at a density of 1.0 x 10^6^ cells/well. The cells were cultured for 24 hours in HEK-293T growth medium (DMEM containing 10% fetal bovine serum, 2 mM L-glutamine, 1 mM sodium pyruvate, 100 U/mL penicillin, and 0.1 mg/mL streptomycin) and then transfected as follows. pBMN vectors containing the appropriate ARHGAP36 construct (1.33 μg) and the pCL-ECO retrovirus packaging vector (0.67 μg) were diluted in OMEM medium (75 μL), and the solution was added to OMEM (75 μL) containing 6 μL Fugene HD reagent (Promega). The mixture was incubated at room temperature for 10 minutes and gently added to the growth medium on the cultured cells. After 24 hours, the medium was replaced with DMEM containing 1.8 mM L-glutamine, 4% fetal bovine serum, 6% calf serum, 1 mM sodium pyruvate, 100 U/mL penicillin, and 0.1 mg/mL streptomycin. Retrovirus-containing supernatant was then collected two times at 20-hour intervals, passed through a 0.45-μm filter, and stored at −80°C. Large-scale retrovirus production was conducted using HEK-293T cells seeded on 10-cm plates at a density of 5.0 x 10^6^ cells/well transfected with 6.48 μg of the ARHGAP36 construct, 4 μg of pCL-ECO, and 35 μL of Fugene HD in 750 μL OMEM medium.

### Generation of ARHGAP36-expressing cell lines

NIH-3T3 or SHH-LIGHT2 cells were seeded into individual wells of 24-well or 6-well plates at a density of 7.5 x 10^4^ or 2.0 x 10^5^ cells/well, respectively. The cells were cultured for 24 hours in either NIH-3T3 growth medium (DMEM containing 10% calf serum, 1 mM sodium pyruvate, 100 U/mL penicillin, and 0.1 mg/mL streptomycin) or SHH-LIGHT2 growth medium (NIH-3T3 growth medium containing 150 μg/mL zeocin and 400 μg/mL G418) and then transduced with 4 μg/mL polybrene and retrovirus for the appropriate ARHGAP36-3xFLAG-IRES-mCherry construct to achieve a multiplicity of infection (MOI) < 0.8. After 24 hours, the medium was exchanged, and the cells were expanded for fluorescence-activated cell sorting (FACS).

For FACS, the cells were washed with PBS buffer, dissociated with TrypLE (Invitrogen) for 3–5 minutes at 37°C, and centrifuged at 106 *g* for 5 minutes at 4°C. Cell pellets were then resuspended in FACS buffer (PBS containing 1% calf serum), passed through a 70-μm cell strainer (BD Biosciences), and added to round-bottom FACS tubes. Cell populations with comparable mCherry fluorescence intensities were then obtained using one of the following sorters: BD FACSAria II (532-nm laser and 600-nm longpass filter, or 561-nm laser and 610/20-nm bandpass filter), BD Influx (561-nm laser and 610/20-nm bandpass filter), or BD FACSAria Fusion (561-nm laser, 600-nm longpass filter, and 610/20-nm bandpass filter).

### Quantitative reverse transcription-PCR (qRT-PCR) analyses

NIH-3T3 cell lines expressing the indicated FLAG-tagged ARHGAP36 constructs were seeded into 6-well plates at a density of 5.2 x 10^5^ cells/well and cultured in NIH-3T3 growth medium. An uninfected condition was also prepared as a negative control. After two days, fully confluent cells were treated with NIH-3T3 low-serum medium (DMEM containing 0.5% calf serum, sodium pyruvate and antibiotics) with or without 10% SHH-N-conditioned medium for 30 hours. The media was replaced with ice-cold PBS, and the cells were collected by manual scraping. Each resulting cell suspension was divided into two tubes (one each for qRT-PCR and western blot analyses) and centrifuged at 750 *g* for 7 minutes at 4°C.

Cell pellets were prepared for qRT-PCR analyses as follows. RNA was isolated using the Monarch Total RNA miniprep kit (New England Biolabs), and equivalent amounts of RNA were used to synthesize cDNA using the SuperScript III First-Strand Synthesis System (Invitrogen). qRT-PCR was performed on a Lightcycler 480 II (Roche) using the following TaqMan probes: Gli1-Mm00494645_m1, Beta-2-Microglobulin-Mm00437762_m1 (Applied Biosystems). Gene expression levels were normalized to β-2-microglobulin. For ARHGAP36 truncation mutant and *Prepl/Praja2* overexpression analyses, the normalized gene expression levels were compared to that of control cells, and for point mutant analyses, compared to that of wild-type ARHGAP36. The resulting gene expression levels were averaged across three biological replicates, and *P* values were determined using either a Student’s one-tailed t-test (ARHGAP36 truncation mutant analyses) or two-tailed t-test (*Prepl/Praja2* overexpression and ARHGAP36 point mutant analyses).

### Western blot analyses

Cell pellets were resuspended in Laemmli sample buffer (10% glycerol, 2% SDS, 17 mM DTT, 0.01% bromophenol blue, 60 mM Tris-HCl (pH 6.8), and protease and phosphatase inhibitors (Roche)). After incubation for 20 minutes at 4°C, cell lysates were boiled for 10 minutes and sonicated in a water bath for 15 seconds. Equivalent amounts of total protein per lysate were loaded onto Criterion XT 4–12% Bis-Tris polyacrylamide gels (Bio-Rad), transferred onto PVDF membranes (Bio-Rad), and detected using the antibodies listed in S1 Table with either SuperSignal West Dura or SuperSignal Femto kits (Pierce) and a ChemiDoc XRS imaging system (Bio-Rad). Band intensities were quantified using ImageLab software (Bio-Rad) and normalized to KPNB1 levels in the corresponding sample. For each replicate, the normalized band intensity in each condition was normalized to that of uninfected cells (PKA_cat_ measurements) or of the indicated ARHGAP36 isoform (FLAG-tagged protein measurements). The resulting relative band intensities for each condition was averaged across three biological replicates, and *P* values were determined using a Student’s two-tailed t-test.

### Immunofluorescence studies

The subcellular localizations of FLAG-tagged ARHGAP36 constructs were assessed as follows. NIH-3T3 cells were seeded onto individual wells of a 6-well plate at a density of 2.0 x 10^5^ cells/well. Cells were cultured for 24 hours in NIH-3T3 growth medium, then transduced with 4 μg/mL polybrene and retrovirus for the appropriate ARHGAP36-3xFLAG-IRES-mCherry construct to achieve an MOI < 0.5. After 24 hours, cells were re-seeded at a 1:8 dilution into 24-well plates containing poly-D-lysine-coated 12-mm glass coverslips and cultured for 1–2 days in growth medium. Cells were fixed in PBS containing 4% paraformaldehyde for 10 minutes at room temperature and washed 3 times with PBS. Cells were next permeabilized with PBS containing 0.5% Triton X-100 for 5 minutes, washed 2 times with PBS, and incubated in blocking buffer (PBS containing 1% BSA and 0.1% Triton X-100) for 1 hour at room temperature. The cells were then incubated for 1 hour at room temperature with primary antibodies diluted in blocking buffer, washed 4 × 5 minutes with PBS containing 0.1% Triton X-100, incubated for 1 hour with the appropriate secondary antibodies diluted in PBS containing 0.2% Triton X-100, and washed 4 × 5 minutes with PBS. The coverslips were rinsed briefly in water and mounted onto slides using Prolong Gold Antifade reagent with DAPI (Invitrogen).

To determine the subcellular localizations of mCherry-tagged ARHGAP36 isoform 3 point mutants, SHH-EGFP cells were seeded into individual wells of a 24-well plate at a density of 7.5 x 10^4^ cells/well. The cells were cultured for 24 hours in SHH-EGFP growth medium (NIH-3T3 growth medium containing 150 μg/mL zeocin) for 24 hours and then transduced with 4 μg/mL polybrene and retrovirus for the appropriate ARHGAP36-mCherry construct to achieve an MOI < 0.5. After 24 hours, the cells were passaged into a new 24-well plate at a 1:1.5 dilution and cultured in growth medium for an additional 2 days to achieve 100% confluency. Confluent cells were then treated for 24 hours with SHH-EGFP low-serum medium (DMEM containing 0.5% calf serum, sodium pyruvate, zeocin, and antibiotics). Cells were next passaged at a 1:3 dilution into 24-well plates containing poly-D-lysine-coated 12-mm glass coverslips and cultured for 1 day in growth medium. Cells were then fixed, blocked, immunostained, and mounted as described above. The subcellular localizations of mCherry-tagged isoform 2 constructs were similarly assessed, with the exception that the cells were passaged onto coverslip-containing 24-well plates 24 hours after retroviral transduction then cultured for two days prior to being fixed.

The effects of ARHGAP36 isoforms on PKA_cat_ localization were assessed as follows. NIH-3T3 cell lines stably expressing FLAG-tagged ARHGAP36 isoforms 2 or 3 were seeded into 24-well plates containing poly-D-lysine-coated 12-mm glass coverslips at a density of 1.2 x 10^5^ cells/well. Cells were then cultured in NIH-3T3 growth medium for 2 days, at which time the cells were fixed in PBS containing 2% paraformaldehyde for 20 minutes at room temperature and then treated with methanol for 5 minutes at –20°C. The fixed cells were incubated in blocking buffer for 1 hour at room temperature and incubated with primary antibodies diluted in blocking buffer overnight at 4°C. Subsequent PBS washes, secondary antibody incubation, and mounting were conducted as described above.

Fluorescence images were obtained using either a Zeiss LSM 700 or 800 confocal microscope equipped with a 63x oil-immersion objective. Maximum-intensity Z-stack projections were created using either ZEN Black (Zeiss), ZEN Blue (Zeiss), or FIJI [59] software, fluorescence intensities were adjusted using FIJI, and images were cropped using Photoshop CC (Adobe).

### Mutant library generation

A library encoding ARHGAP36 isoform 2 mutants was created via error-prone PCR (epPCR) using the GeneMorph II Random Mutagenesis Kit (Agilent). To determine the optimal epPCR conditions for library generation, the mutation frequency was estimated for epPCRs consisting of 15, 20, 25, or 29 cycles. All reactions were conducted according to the manufacturer’s instructions, using 1.64 μg of the pBMN-ARHGAP36 (isoform2)-mCherry plasmid as template, 0.4 μM of each primer (S1 Table; primers 49 and 70), and 4% DMSO. The product yield for each condition was estimated by resolving 10% of the reaction on a 1% EtBr-agarose gel and quantifying the band intensity of the resulting amplicon with ImageLab software (Bio-Rad). The 1.6-kb amplicon was gel-extracted using the QiaQuick Gel Extraction Kit (Qiagen) and ligated into a XhoI- and SacII-digested pBMN-ARHGAP36 (isoform 2)-mCherry vector using Gibson assembly. To estimate the mutation frequency for each epPCR-generated library, XL-10 Gold *E*. *coli* (Agilent) were chemically transformed with 1:4 diluted Gibson assembly products and plated onto ampicillin-agarose plates. Forty colonies from each plate were sequenced using rolling circle amplification and Sanger sequencing (Sequetech) (S1 Table; primers 71–76). High-quality *ARHGAP36* reads were aligned to the coding sequence for wild-type ARHAGP36 isoform 2, and the number of nucleotide mutations within the coding sequence was counted for each read. This analysis yielded the distribution of mutated nucleotides across the library.

Through these pilot studies, we found that the 15-cycle epPCR conditions maximized the percentage of *ARHGAP36* variants with single-nucleotide changes. We next generated a large-scale library using the 15-cycle epPCR and Gibson assembly strategy described above. The undiluted Gibson reaction (8 μL) was electroporated into 160 μL of MegaX DH10B T1^R^ Electrocomp cells (Invitrogen). The electroporated cells were immediately transferred to 480 mL of Superior Broth containing 75 μg/mL ampicillin, and 100 μL of the culture was plated on ampicillin-agar plates to estimate the number of colony-forming units. The final library was found to contain approximately 4 x 10^5^ colony-forming units, which corresponds to an equivalent number of library elements. The liquid culture was incubated at 30°C until it reached an OD_600_ of 1, after which plasmids were isolated using the NucleoBond Xtra Midi Plus Kit (Macherey-Nagel).

Retroviral medium harboring the ARHGAP36 mutant library was generated in the following manner. One 10-cm plate of HEK-293T cells at 90% confluency was transfected with 6.5 μg of the pBMN-ARHGAP36 (isoform 2)-mCherry mutant library and 4 μg of pCL-ECO using the FuGene HD transfection reagent (Promega). The medium was replaced after 24 hours with DMEM containing 1.8 mM L-glutamine, 4% fetal bovine serum, 6% calf serum, 1% sodium pyruvate, 100 U/mL penicillin, and 0.1 mg/mL streptomycin. Retrovirus-containing supernatant was then collected two times at 24-hour intervals, passed through a 0.45-μm filter, and stored at −80°C.

### FACS-based screening

SHH-EGFP cells were seeded onto a 15-cm plate at a density of 1.0 x 10^6^ cells/plate and cultured in SHH-EGFP growth medium for 2 days, then transduced with the retroviral library of ARHGAP36 mutants and 4 μg/mL polybrene to achieve an MOI < 0.5. After 24 hours, the cells were expanded to 4 x 15-cm plates and cultured for an additional 2 days. SHH-EGFP cells treated with 10% SHH-N-conditioned media or transduced with retrovirus encoding wild-type ARHGAP36 isoform 2 served as positive controls for flow cytometry. For negative controls, untreated SHH-EGFP cells or cells transduced with retrovirus encoding ARHGAP36 isoform 1 were used.

To isolate mCherry+ cells by FACS, the transduced SHH-EGFP cells were washed with PBS, dissociated with TrypLE (Invitrogen) for 3–5 minutes at 37° C, and centrifuged at 750 *g* for 7 minutes at 4°C. The resulting cell pellets were resuspended in FACS buffer (PBS containing 1% calf serum), passed through a 70-μm cell strainer, and added to round-bottom FACS tubes. Cell sorting was performed on a BD FACSAria II configured with a 561-nm laser, a 595-nm longpass filter, and a 616/23-nm bandpass filter for mCherry detection. Data was collected with FACSDiva software (BD Biosciences) and analyzed using FlowJo (FlowJo). 2.7 x 10^7^ cells were analyzed by FACS analysis.

This first sort produced a population of 3 x 10^6^ mCherry+ cells, which were cultured for 3 days until they reached 100% confluency. The cells were then cultured in SHH-EGFP low-serum medium to promote primary cilium formation and enable ARHGAP36-mediated Gli activation. After 24 hours, cells were expanded at a 1:2 dilution, cultured for 24 hours to achieve full confluency, and serum-starved again for another 24 hours.

To isolate cells expressing inactive forms of ARHGAP36 isoform 2 (mCherry+/EGFP–), 1.1 x 10^7^ cells from the expanded mCherry+ population were washed and dissociated as described above. Approximately 3 x 10^6^ cells were separated and expanded to 4 x 10^7^ cells to establish a pre-selection population, which was then washed with PBS, dissociated, and pelleted by centrifugation at 750 *g* for 7 minutes at 4°C. The pellet was stored at –80°C until used for genomic DNA extraction. The remaining 8 x 10^6^ mCherry+ cells were analyzed by FACS to select for those expressing inactive ARHGAP36-mCherry mutants. Cells were sorted as described above using the BD FACSAria II configured with a 488-nm dye laser, a 495-nm longpass filter, and a 530/30-nm bandpass filter for EGFP detection and the laser/filter configurations described above for mCherry detection. Approximately 4 x 10^5^ mCherry+/EGFP–cells were obtained from this second sort, and they were cultured for 2 days to reach full confluency and then subjected to 2 rounds of serum starvation. FACS sorting of this enriched population yielded 1.6 x 10^5^ mCherry+/EGFP–cells, which were expanded, frozen in SHH-EGFP growth medium containing 10% DMSO, and stored in liquid nitrogen.

We then assessed if the mCherry+/EGFP–cells were still capable of Gli-dependent EGFP expression under canonical Hh pathway activation conditions. Frozen aliquots of cells from the third sort were thawed, expanded, and subjected to two rounds of serum starvation. Approximately 1.0 x 10^7^ cells were sorted with a BD FACSAria IIu configured with a 488-nm laser, a 502-nm longpass filter, and a 525/50-nm bandpass filter for EGFP detection and a 488-nm laser, a 595-nm longpass filter, and a 610/20-nm bandpass filter for mCherry detection. 1.0 x 10^6^ mCherry+/EGFP–cells were collected and cultured for 7 days to achieve full confluency. The cells were then treated with 200 nM SAG in SHH-EGFP low-serum medium for 24 hours, expanded at a 1:2 dilution, cultured for 24 hours to enable full confluency, and treated again with 200 nM SAG for 24 hours. 2 x 10^7^ cells were then sorted with the BD FACSAria IIu to obtain 3 x 10^6^ mCherry+/EGFP+ cells. This selected population was expanded to 5 x 10^6^ cells, which were then washed with PBS, dissociated, and pelleted by centrifugation at 750 *g* for 7 minutes at 4°C. The pellet was stored at –80°C until used for genomic DNA extraction.

### Deep-sequencing analyses of pre- and post-selection pools

Genomic DNA was extracted from frozen pellets using the QIAamp DNA Blood Maxi Kit (Qiagen) according to manufacturer’s instructions. For each sample, *ARHGAP36* inserts were isolated from genomic DNA by PCR using 500 ng of genomic DNA, 0.5 μM each of primer (S1 Table; primers 77–78), 0.2 μM dNTP mix, and Phusion High-Fidelity DNA Polymerase in HF buffer (New England Biolabs). A total of 182 PCRs were used to isolate *ARHGAP36* inserts from 91 μg of pre-selection genomic DNA, while 89 PCRs were used to isolate inserts from 45 μg of post-selection genomic DNA. For each condition, the respective reactions were pooled, purified using the QIAquick PCR Purification Kit (Qiagen), and resolved on a 0.8% EtBr–agarose gel. The 1.6-kb amplicon was then gel-extracted using the QIAquick Gel Extraction Kit (Qiagen). Amplicons were quantified using a Bioanalyzer 2100 with high-sensitivity DNA kits (Agilent), sheared into 150-bp fragments with an S220 focused-ultrasonicator (Covaris), and sequenced on a NextSeq 500 Sequencer using High-Output v2 kits (Illumina).

FASTQ files were aligned to the wild-type *ARHGAP36* coding sequence (Bowtie2 v2.3) [60] and sorted by read name (SAMtools v1.3.1) [61]. The resulting mapped and sorted reads were then analyzed with an in-house Python script. Briefly, high-quality reads were aligned to the wild-type *ARHGAP36* coding sequence. Reads with greater than 3 high-quality mutations or with internal stop codons were discarded from further analysis. The remaining reads were translated, identifying the ARHGAP36 amino acid mutations present in the population. For each residue, the fold-change in its mutation frequency between the pre- and post-selection populations was calculated.

### Flow cytometry-based assays

SHH-EGFP cells were seeded into individual wells of a 24-well plate at a density of 7.5 x 10^4^ cells/well and cultured for 24 hours in SHH-EGFP growth medium. The cells were then transduced with retrovirus for the appropriate ARHGAP36-mCherry construct and 4 μg/mL polybrene to achieve an MOI < 0.5. An uninfected condition was also prepared as a negative control. After 24 hours, the cells were passaged onto a new 24-well well at a 1:1.5 dilution and cultured in growth medium for an additional 2 days to achieve 100% confluency. Confluent cells were then treated with SHH-EGFP low-serum medium for 24 hours. Cells were then passaged at a 1:1.5 dilution onto a new 24-well well and cultured for 24 hours to achieve full confluency for a second round of 24-hour serum-starvation with or without SHH-N-conditioned medium.

For flow cytometry analyses, the cells were washed with PBS, dissociated with TrypLE for 3–5 minutes at 37°C, and centrifuged 750 *g* for 7 minutes at 4°C. Cell pellets were resuspended in FACS buffer (PBS containing 1% calf serum) and analyzed on a DxP FACScan (561-nm laser and 616/25-nm bandpass filter for mCherry detection; 488-nm laser, 560-nm shortpass filter, and 525/50-nm bandpass filter for EGFP detection) or a BD LSRII (561-nm laser, 600-nm longpass band filter, and a 610/20-nm bandpass filter for mCherry detection; 488-nm laser, 505-nm longpass band filter, and 525/50-nm bandpass filter for EGFP detection). Data was collected with Cypod (Cytek) and FACSDiva software and analyzed using FlowJo. Fluorescence data was collected for at least 2.5 x 10^4^ cells, and three biological replicates were analyzed for each condition.

Data analyses excluded mCherry–cells, which is indicative of a lack of ARHGAP36 expression. For each replicate, the Gli activity in each condition was measured by calculating the percentage of cells that exhibited EGFP fluorescence comparable to that of cells expressing wild-type ARHGAP36. This value was then normalized to that of wild-type ARHGAP36-expressing cells, and a Student’s one-tailed t-test was used to identify mutations that significantly altered the percentage of EGFP-expressing cells (*P* ≤ 0.05).

### Tandem affinity purification and quantitative proteomics

NIH-3T3 cells were seeded onto 15-cm plates (6 per condition) at a density of 2 x 10^6^ cells/plate and cultured in NIH-3T3 growth medium. After 24 hours, cells were transduced with retrovirus for either wild-type or L317P ARHGAP36 with a C-terminal LAP tag and 4 μg/mL polybrene to achieve an MOI > 1.5. The media was exchanged for growth medium after 4 hours, and cells were cultured for an additional 20 hours. Cells were then washed with cold PBS, manually scraped off each dish, and transferred into Falcon tubes. Cell suspensions expressing the same ARHGAP36 construct were combined, and 0.5% of the resulting pool was reserved for downstream flow cytometry analyses to confirm the MOI. The remaining cells were centrifuged at 750 *g* for 7 minutes at 4°C. The supernatant was aspirated, and the remaining cell pellet was flash frozen in liquid nitrogen and stored at –80°C prior to LAP-tagged mediated tandem affinity purification. Three biological replicates were conducted for each wild-type and L317P ARHGAP36-LAP comparison.

Tandem affinity purifications and mass spectrometry analyses were conducted as described previously [32–37]. Pellets of ARHGAP36-LAP-expressing cells were re-suspended in LAP resuspension buffer (300 mM KCl, 50 mM HEPES-KOH (pH 7.4), 1 mM EGTA, 1 mM MgCl_2_, 10% glycerol, 0.5 mM DTT, and protease inhibitors (Thermo Scientific)). Cells were lysed with the gradual addition of 10% NP-40 to a final concentration of 0.3%, followed by a 10-minute incubation at 4°C. The lysate was then centrifuged at 27,000 *g* at 4°C for 10 minutes, and the resulting supernatant was centrifuged at 100,000 *g* for 1 hour at 4°C. The high-speed supernatant was next incubated with anti-GFP-antibody-coupled beads [32–37] for 1 hour at 4°C to capture GFP-tagged proteins. The beads were washed five times with LAP200N buffer (200 mM KCl, 50 mM HEPES-KOH (pH 7.4), 1 mM EGTA, 1 mM MgCl_2_, 10% glycerol, protease inhibitors, and 0.05% NP40) and incubated with PreScission protease in LAP200N buffer at 4°C for 16 hours. All subsequent steps were performed in a laminar flow hood. PreScission protease-eluted supernatant was added to S-protein agarose beads (EMD Millipore) and incubated rocking for 3 hours at 4°C. S-protein agarose beads were then washed three times with LAP200N buffer and twice with LAP100 buffer (100 mM KCl, 50 mM HEPES-KOH (pH 7.4), 1 mM EGTA and 10% glycerol). Beads were stored in 50mM HEPES (pH 7.5), 1 mM EGTA, 1 mM MgCl_2_, 10% glycerol at 4°C prior to on-bead digestion.

Proteins were eluted from S-protein agarose beads with an on-bead reduction, alkylation, and tryptic digestion as follows. Samples were reduced with 10 mM DTT in ammonium bicarbonate for an initial 5-minute incubation at 55°C followed by 25 minutes at room temperature. The proteins were then alkylated with a 30-minute incubation in 30 mM acrylamide at room temperature, and finally eluted from the beads with an overnight digest performed at room temperature using Trypsin/LysC (Promega) and 0.02% ProteaseMax (Promega). The digests were acidified with 1% formic acid, de-salted with C18 Monospin reversed phase columns (GL Sciences), dried on a SpeedVac, and reconstituted in 12.5 μL of 2% acetonitrile and 0.1% formic acid. 4 μL of each sample were used for liquid-chromatography-mass spectrometry analyses performed on an Acquity M-Class UPLC (Waters Corporation) and either an Orbitrap Q-Exactive HFX mass spectrometer (Thermo Scientific) or an Orbitrap Fusion Tribrid mass spectrometer (Thermo Scientific). For each biological replicate, the sample from the ARHGAP36 L317P-expressing cells was run immediately before that of the wild-type ARHGAP36-expressing cells. Analysis of the resulting. RAW data files was conducted using Byonic (Protein Metrics), with the assumption of tryptic proteolysis and a maximum allowance of two missed cleavage sites. Precursor and MS/MS fragment mass accuracies were held within 12 ppm and 0.4 Da, respectively. A false discovery rate of 1% was used for protein identification [62].

The resulting list of identified proteins was compared to an NCBI FASTA database containing all mouse proteomic isoforms with the exception of the tandem affinity bait construct sequence and common contaminant proteins. Post-processing of spectral counts was conducted with an in-house R script. For each protein, spectral counts detected across all isoforms were combined and normalized to the mean amino acid length of all isoforms. The resulting normalized spectral count was divided by the sum of normalized spectral counts calculated for all proteins in the pulldown sample, generating a normalized spectral abundance factor (NSAF) for each protein. To account for variability in bait ARHGAP36 expression across different pulldown samples, the NSAF of each protein was divided by that of the bait ARHGAP36 (relative NSAF). Proteins that were detected in all biological replicates for a given condition were tabulated, resulting in a dataset of 566 candidate ARHGAP36-binding proteins. For each protein, the fold-change in relative NSAF between the wild-type and L317P mutant ARHGAP36 pulldown samples of the same replicate was calculated. The average fold-change in relative NSAF across of all three replicates was then calculated for each protein.

To assess the robustness of our comparative interactome analyses, we calculated a modified Z score that compares the protein enrichment in either interactome (wild-type vs. L317P ARHGAP36) against the experimental variability in protein abundance measurements. Protein enrichment was represented by the log_2_-transformation of the average fold change in relative NSAF between wild-type and L317P ARHGAP36 interactomes [log_2_ (WT NSAF:L317 NSAF)]. We estimated the error in measuring protein abundance in a given interactome by normalizing the relative NSAFs for each replicate to the average value across all three replicates (mean-normalized relative NSAFs). This transformation approximates how much the variation between replicates can contribute to an apparent fold change. To place equal weight on upward and downward variations from the mean, we calculated the absolute value of the log_2_-transformed mean-normalized relative NSAFs. These calculations were conducted for the relative NSAFs of a protein in both the wild-type and L317P mutant ARHGAP36 interactomes, and the two resulting values were summed to produce a final error estimate. The modified Z score of each protein was calculated by dividing the log_2_ (WT NSAF:L317 NSAF) by the final error estimate and then calculating the absolute value of the resulting quotient. Proteins with higher modified Z scores are those that are enriched in a given interactome to a degree that is greater than the estimated experimental error.

### *Prepl*/*Praja2* siRNA studies

Untransduced and ARHGAP36-expressing SHH-LIGHT2 cells were transfected with pooled siRNAs (Dharmacon) against *Gli2* (L-043977-01-0005), *Praja2* (L-058919-01-0005), *Prepl* (L-059506-01-0005), or a non-targeting control (D-001810-01-05) as follows. siRNAs were diluted in 190 μL OMEM to a final concentration of 10 nM, and the solution was added to OMEM (190 μL) containing 10 μL Dharmafect3 (Dharmacon). This mixture was incubated for 30 minutes in a 6-well plate, after which the cells were added at a density of 3.2 x 10^5^ cells/well in SHH-LIGHT2 growth medium. The cells were cultured for 48 hours, at which time the fully confluent cells were treated for 30 hours with SHH-LIGHT2 low-serum medium (DMEM containing 0.5% calf serum sodium pyruvate, zeocin, G418, and antibiotics) with or without 200 nM SAG. The cells were then washed with PBS, dissociated with TrypLE for 3–5 minutes at 37° C, and resuspended in SHH-LIGHT growth media. Each resulting cell suspension was divided into two tubes (one each for luciferase and western blot analyses) and centrifuged at 750 *g* for 7 minutes at 4°C.

Luciferase activities were measured in cell lysates using a Dual-Luciferase Reporter Assay System (Gold Biotechnology) on a Veritas luminometer (Turner BioSystems). Gli-dependent firefly luciferase activity was normalized to that of constitutively active *Renilla* luciferase. Normalized Gli-dependent luciferase activities were compared to that of untransduced SHH-LIGHT2 cells transfected with a non-targeting control siRNA. The resulting values were averaged across six biological replicates, and *P* values were determined using a Student’s two-tailed t-test.

### *Prepl*/*Praja2* overexpression studies

NIH-3T3 cell lines expressing the indicated FLAG-tagged ARHGAP36 constructs were seeded into individual wells of 6-well plates at a density of 2.0 x 10^5^ cells/well. The cells were cultured for 24 hours in NIH-3T3 growth medium and then transduced with 4 μg/mL polybrene and retrovirus for either PREPL-3xFLAG-IRES-TagBFP or PRAJA2-3xFLAG-IRES-TagBFP constructs to achieve an MOI > 0.5. Cells transduced with a 3xFLAG-IRES-mCherry construct served as negative controls. The medium was exchanged after 24 hours, and cells were cultured for an additional 24 hours in NIH-3T3 growth medium. The fully confluent cells were then treated with NIH-3T3 low-serum medium with or without 200 nM SAG for 30 hours. The cells were then washed with PBS, dissociated with TrypLE for 3–5 minutes at 37° C, and resuspended in NIH-3T3 growth media. Each resulting cell suspension was divided into two tubes (one each for qRT-PCR and western blot analyses) and centrifuged at 750 *g* for 7 minutes at 4°C.

### Co-immunoprecipitation studies

To determine interactions between the N3 and GAP-like domains, HEK-293 cells were seeded into three individual wells of a 6-well plate at a density of 1.0 x 10^6^ cells/well, cultured for 24 hours in HEK-293T growth medium, and transfected as described with Fugene HD reagent (7 μL/well), OMEM medium (68 μL/well), and pBMN vectors encoding N3-3xFLAG-IRES-mCherry (1 μg/well) and either pBMN-GFP or pBMN-GAP-LAP (1 μg/well). Cells were then washed with cold PBS, manually scraped off each dish, and centrifuged at 750 *g* for 7 minutes at 4°C. The supernatant was aspirated, and the remaining cell pellet was resuspended in NP-40 lysis buffer (1% NP-40, 20 mM Tris-HCl (pH 7.5), 150 mM NaCl, 1 mM EGTA, 5 mM NaF, 25 mM N-ethylmaleimide (Sigma), and protease and phosphatase inhibitors (Roche)) and incubated at 4°C rotating end-over-end for 30 minutes. Samples were then centrifuged at 15,000 *g* at 4°C for 10 minutes, and the resulting supernatant was collected as cell lysate. Lysates were normalized to a total protein concentration of 0.4 mg/mL. For immunoprecipitation, 96 μL of Protein G Dynabeads (Invitrogen) were washed three times for 5 minutes in 240 μL of wash buffer (0.3% NP-40, 20 mM Tris-HCl (pH 7.5), 150 mM NaCl, 1 mM EGTA, 5 mM NaF) and incubated with 14.4 μg of mouse IgG2a anti-GFP antibodies (Invitrogen) in 384 μL wash buffer at 4°C rotating end-over-end for two hours. After three five-minute washes, the antibody-conjugated beads were resuspended in 240 μL of wash buffer and 100 μL of the mixture was distributed to each of two tubes. The wash buffer was replaced with 1.8 mL of lysate, and the suspension was incubated at 4°C rotating end-over-end overnight to capture GFP-tagged proteins. The beads were then washed five times for 15 minutes at 4°C and boiled in 200 μL of Laemmli sample buffer for 10 minutes to elute GFP-tagged proteins and interactors. Samples were sonicated in a water bath for 15 seconds prior to western blot analyses. Input samples were similarly generated with 35 μL of diluted lysate and 7 μL of 6X Laemmli sample buffer. FLAG- and GFP-tagged protein levels in input and immunoprecipitate samples were measured by western blot as described above.

Interactions between PREPL and select ARHGAP36 constructs were assessed by seeding NIH-3T3 cells onto 10-cm plates at a density of 1 x 10^6^ cells/plate and culturing the fibroblasts in growth medium for 24 hours. The cells were then transduced with retrovirus for ARHGAP36 constructs with a C-terminal FLAG tag and 4 μg/mL polybrene to achieve an MOI > 1. The media was exchanged for growth medium after 4 hours, and cells were cultured for an additional 20 hours. Cells were lysed as described for anti-GFP immunoprecipitation studies, and the lysates were normalized to a total protein concentration of 0.1 mg/mL. For immunoprecipitation of FLAG-tagged proteins, 808 μL of anti-FLAG M2 magnetic bead slurry (Millipore) were washed twice for 5 minutes in 4 mL of wash buffer (20 mM Tris-HCl (pH 7.5), 250 mM NaCl, 1 mM EGTA, 5 mM NaF) prior to resuspension of the packed magnetic resin in 404 μL lysis buffer. For each sample, 200 μL of lysate was incubated with 160 μL of bead suspension at 4°C rotating end-over-end for 1 hour to capture FLAG-tagged proteins. The beads were then washed five times with wash buffer for 15 minutes at 4°C, and FLAG-tagged proteins and interactors were eluted with two rounds of incubation in 250 μL of wash buffer containing 1% NP-40 and 150 ng/μL 3XFLAG peptide (APExBIO) for 1 hour at 4°C. Both eluates were pooled, combined with 100 μL of 6X Laemmli sample buffer, and then boiled for 10 minutes and sonicated in a water bath for 15 seconds prior to western blot analyses. Input samples were similarly generated with 35 μL of diluted lysate and 7 μL of 6X Laemmli sample buffer.

PREPL and FLAG-tagged protein levels in input and immunoprecipitate samples were measured by western blot as described above. PREPL band intensities in immunoprecipitates were normalized to FLAG-tagged protein levels in the corresponding sample. For each replicate, the normalized band intensity in each condition was normalized to that of cells expressing ARHGAP36 isoform 2. The resulting relative band intensities for each condition was averaged across at least three biological replicates, and *P* values were determined using a Student’s two-tailed t-test.

### Statistical analyses

Biological replicates are defined as experimental samples that are capable of biological variance, and technical replicates are defined as those for which experimental variance is solely dependent on measurement accuracy.

## Supporting information

S1 Fig The C212Y mutation attenuates the ability of ARHGAP36 isoform 2 to activate Gli function. (PDF)

S2 Fig ARHGAP36 isoforms differentially induce PKA_cat_ depletion. (PDF)

S3 Fig Inactivating point mutations in the GAP-like domain render ARHGAP36 cytosolic. (PDF)

S4 Fig Point mutations in the GAP-like domain attenuate the activity and localization of ARHGAP36 isoform 3. (PDF)

S5 Fig The GAP-like domain can interact with N3. (PDF)

S6 Fig PRAJA2 does not regulate ARHGAP36-mediated Gli activation. (PDF)

S7 Fig ARHGAP36-PREPL binding requires the GAP-like domain. (PDF)

S1 Dataset Mutation frequencies in pre- and post-selection populations. (XLSX)

S2 Dataset Wild-type and L317P ARHGAP36 isoform 2 interactomes. (XLSX)

S1 Table Antibody and primer resources. (XLSX)

S1 Raw images Original western blot images. (PDF)
