# Supplementary material for: Structure-activity mapping of ARHGAP36 reveals regulatory roles for its GAP homology and C-terminal domains
Source: PLoS One. 2021 May 17;16(5):e0251684. doi: 10.1371/journal.pone.0251684 (PMC8128262; doi:10.1371/journal.pone.0251684)
Supplement: S1 Fig — (PDF) [file pone.0251684.s001.pdf]

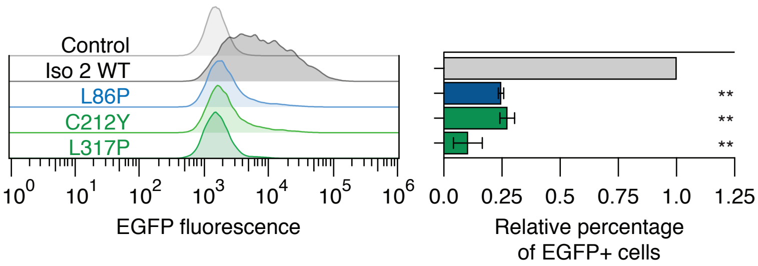

S1 Fig. The C212Y mutation attenuates the ability of ARHGAP36 isoform 2 to activate Gli function. Activities of indicated ARHGAP36-mCherry variants in SHH-EGFP cells, as assessed by flow cytometry. mCherry fluorescence intensities were used to gate cells with comparable levels of ARHGAP36 expression, and the distributions of EGFP fluorescence (left) and relative percentage of EGFP+ cells (right) are shown for each ARHGAP36 construct. Data are the average fold change in the percentage of EGFP+ cells relative to that of cells expressing wild-type ARHGAP36 for three biological replicates ± s.e.m. Double asterisks indicate $P < 0.01$.
