# Supplementary material for: Structure-activity mapping of ARHGAP36 reveals regulatory roles for its GAP homology and C-terminal domains
Source: PLoS One. 2021 May 17;16(5):e0251684. doi: 10.1371/journal.pone.0251684 (PMC8128262; doi:10.1371/journal.pone.0251684)
Supplement: S2 Fig — (PDF) [file pone.0251684.s002.pdf]

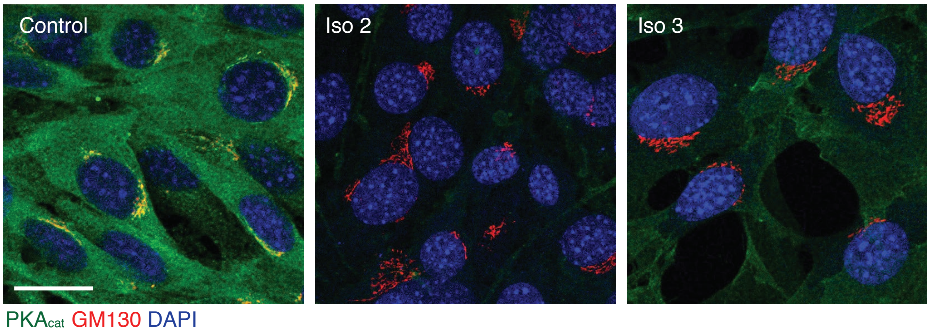

S2 Fig. ARHGAP36 isoforms differentially induce PKA$_{cat}$ depletion. PKA$_{cat}$ localization in NIH-3T3 cells transduced with FLAG-tagged ARHGAP36 isoform 2 or 3. Representative maximum-intensity Z-stack projections are shown with immunofluorescent staining for PKA$_{cat}$, GM130 (cis-Golgi), and DAPI (nucleus). Scale bar: 20 μm.
