# Supplementary material for: Structure-activity mapping of ARHGAP36 reveals regulatory roles for its GAP homology and C-terminal domains
Source: PLoS One. 2021 May 17;16(5):e0251684. doi: 10.1371/journal.pone.0251684 (PMC8128262; doi:10.1371/journal.pone.0251684)
Supplement: S3 Fig — (PDF) [file pone.0251684.s003.pdf]

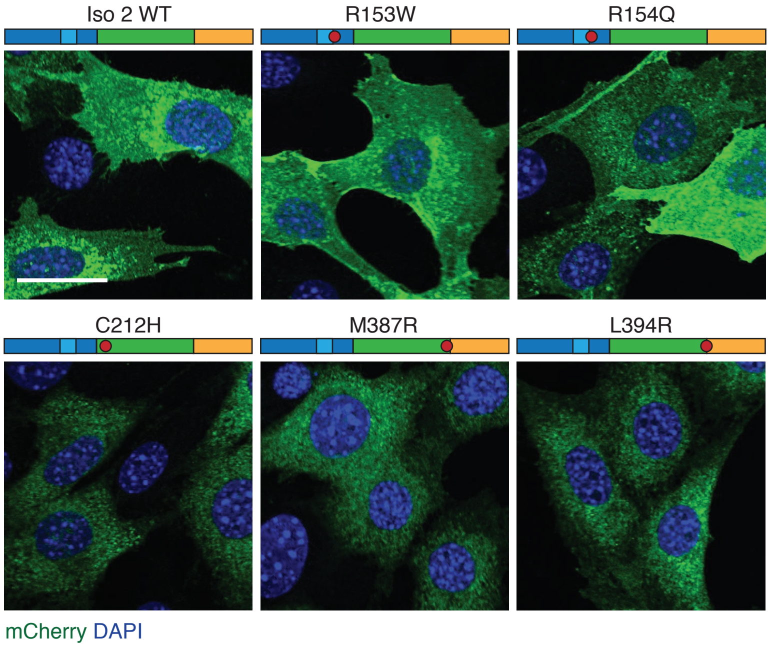

S3 Fig. Inactivating point mutations in the GAP-like domain render ARHGAP36 cytosolic. Representative immunofluorescence micrographs of SHH-EGFP cells retrovirally transduced with the indicated mCherry-tagged ARHGAP36 isoform 2 constructs. Images are maximum-intensity Z-stack projections are shown with immunofluorescent staining for mCherry and DAPI (nucleus). Scale bar: 20 μm.
