# Supplementary material for: Structure-activity mapping of ARHGAP36 reveals regulatory roles for its GAP homology and C-terminal domains
Source: PLoS One. 2021 May 17;16(5):e0251684. doi: 10.1371/journal.pone.0251684 (PMC8128262; doi:10.1371/journal.pone.0251684)
Supplement: S5 Fig — (PDF) [file pone.0251684.s005.pdf]

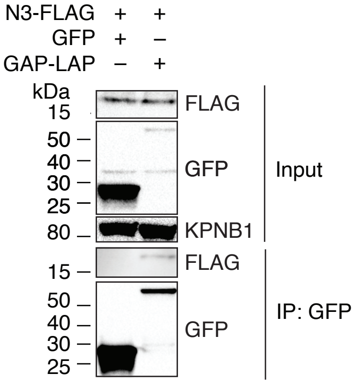

S5 Fig. The GAP-like domain can interact with N3. FLAG-tagged protein levels in anti-GFP immunoprecipitates (IP) of HEK-293 cells co-transfected with FLAG-tagged N3 and either GFP or the GAP-like domain fused to a LAP tag (S-tag-Precission protease site-GFP). Western blots of whole cell lysates for each condition are shown with the importin β1 subunit (KPNB1) used as a loading control.
