# Supplementary material for: Structure-activity mapping of ARHGAP36 reveals regulatory roles for its GAP homology and C-terminal domains
Source: PLoS One. 2021 May 17;16(5):e0251684. doi: 10.1371/journal.pone.0251684 (PMC8128262; doi:10.1371/journal.pone.0251684)
Supplement: S6 Fig — (PDF) [file pone.0251684.s006.pdf]

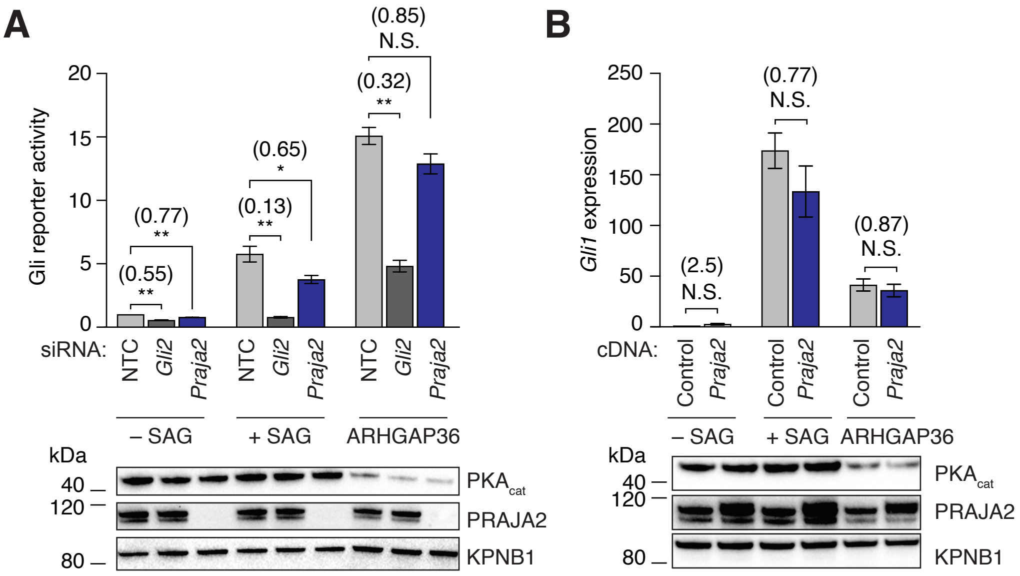

S6 Fig. PRAJA2 does not regulate ARHGAP36-mediated Gli activation. (A) Effects of *Praja2* siRNAs on Gli-dependent luciferase reporter activity in SHH-LIGHT2 cells stimulated with SAG or retrovirally transduced with FLAG-tagged ARHGAP36 isoform 2. SHH-LIGHT2 cells treated with either a non-targeting control siRNA (NTC) or *Gli2* siRNA were included as controls. Data are the average fold change relative to NTC siRNA-transfected, untransduced cells for six biological replicates ± s.e.m. (B) Effects of exogenous *Praja2* expression on *Gli1* transcription in NIH-3T3 cells stimulated with SAG or transduced with FLAG-tagged ARHGAP36 isoform 2. Cells transduced with 3xFLAG-ires-mCherry vector were included as negative controls. Data are the average fold change relative to control vector-transduced, unstimulated cells for three biological replicates ± s.e.m. Values in parentheses represent the fold change relative to cells transfected with NTC siRNA (A) or transduced with control vector (B) within the same treatment group. Single and double asterisks indicate $P < 0.05$ and $P < 0.01$, respectively, and N.S. indicates $P \geq 0.05$. Representative western blots for each condition are shown, with the importin β1 subunit (KPNB1) used as a loading control.
