# Supplementary material for: Structure-activity mapping of ARHGAP36 reveals regulatory roles for its GAP homology and C-terminal domains
Source: PLoS One. 2021 May 17;16(5):e0251684. doi: 10.1371/journal.pone.0251684 (PMC8128262; doi:10.1371/journal.pone.0251684)
Supplement: S7 Fig — (PDF) [file pone.0251684.s007.pdf]

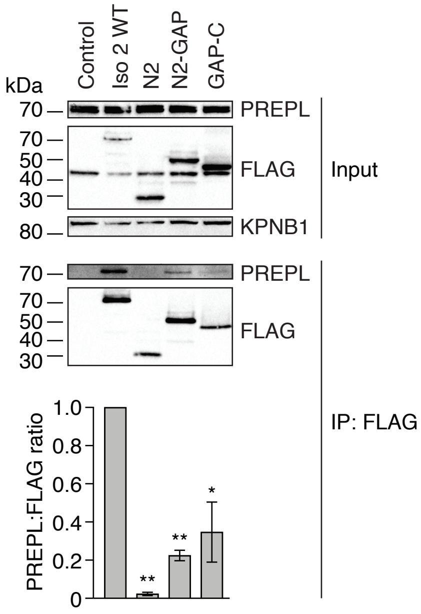

S7 Fig. ARHGAP36-PREPL binding requires the GAP-like domain. PREPL protein levels in anti-FLAG immunoprecipitates (IP) of NIH-3T3 cells retrovirally transduced with the indicated FLAG-tagged ARHGAP36 truncation mutants. Data are the ratio of PREPL and FLAG levels in anti-FLAG immunoprecipitates normalized to that of cells expressing ARHGAP36 isofom 2 (average of three biological replicates ± s.e.m.). Single and double asterisks indicate $P < 0.05$ and $P < 0.01$, respectively. Representative western blots for each condition are shown with the importin β1 subunit (KPNB1) used as a loading control for whole-cell lysate.
