# Supplementary material for: Structure-activity mapping of ARHGAP36 reveals regulatory roles for its GAP homology and C-terminal domains
Source: PLoS One. 2021 May 17;16(5):e0251684. doi: 10.1371/journal.pone.0251684 (PMC8128262; doi:10.1371/journal.pone.0251684)

Figure 2E. Samples are uninfected NIH-3T3 cells treated with or without Hh ligand (Control or Control + Hh, respectively) or NIH-3T3 cells transduced with the indicated FLAG-tagged ARHGAP36 constructs. Lanes not included in the figure are indicated by the red X's. The asterisk indicates WT-expressing samples generated from a different experiment.

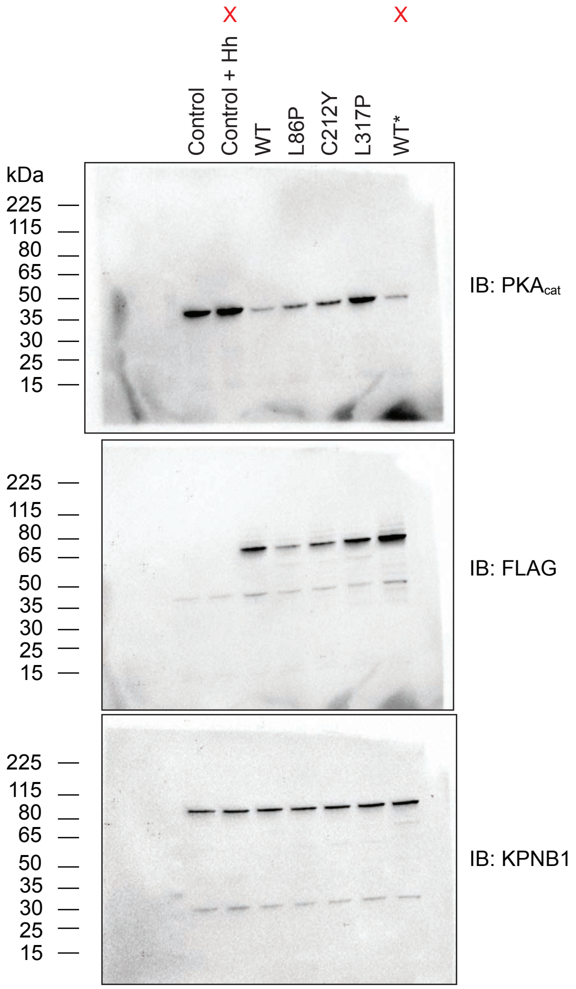

Figure 3B. Samples are uninfected NIH-3T3 cells treated with or without Hh ligand (Control or Control + Hh, respectively) or NIH-3T3 cells transduced with the indicated FLAG-tagged ARHGAP36 constructs. Lanes not included in the figure are indicated by the red X's.

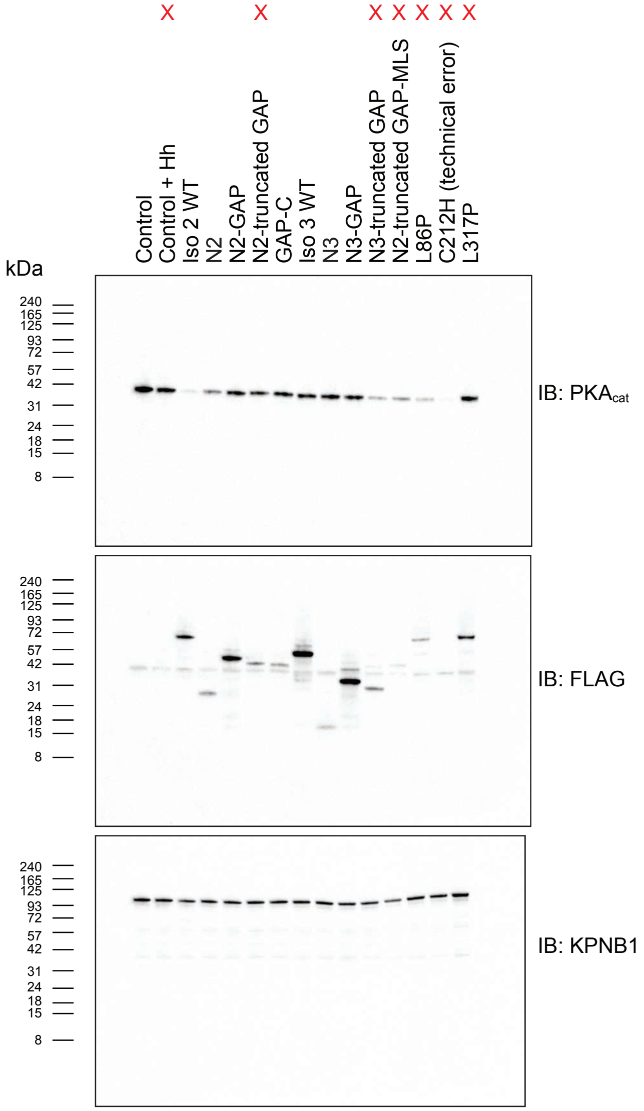

Figure 6A, 6C. Samples are SHH-LIGHT2 cells stimulated with SAG or retrovirally transduced with FLAG-tagged ARHGAP36 isoform 2 and transfected with indicated siRNAs. NTC indicates non-targeting control siRNA.

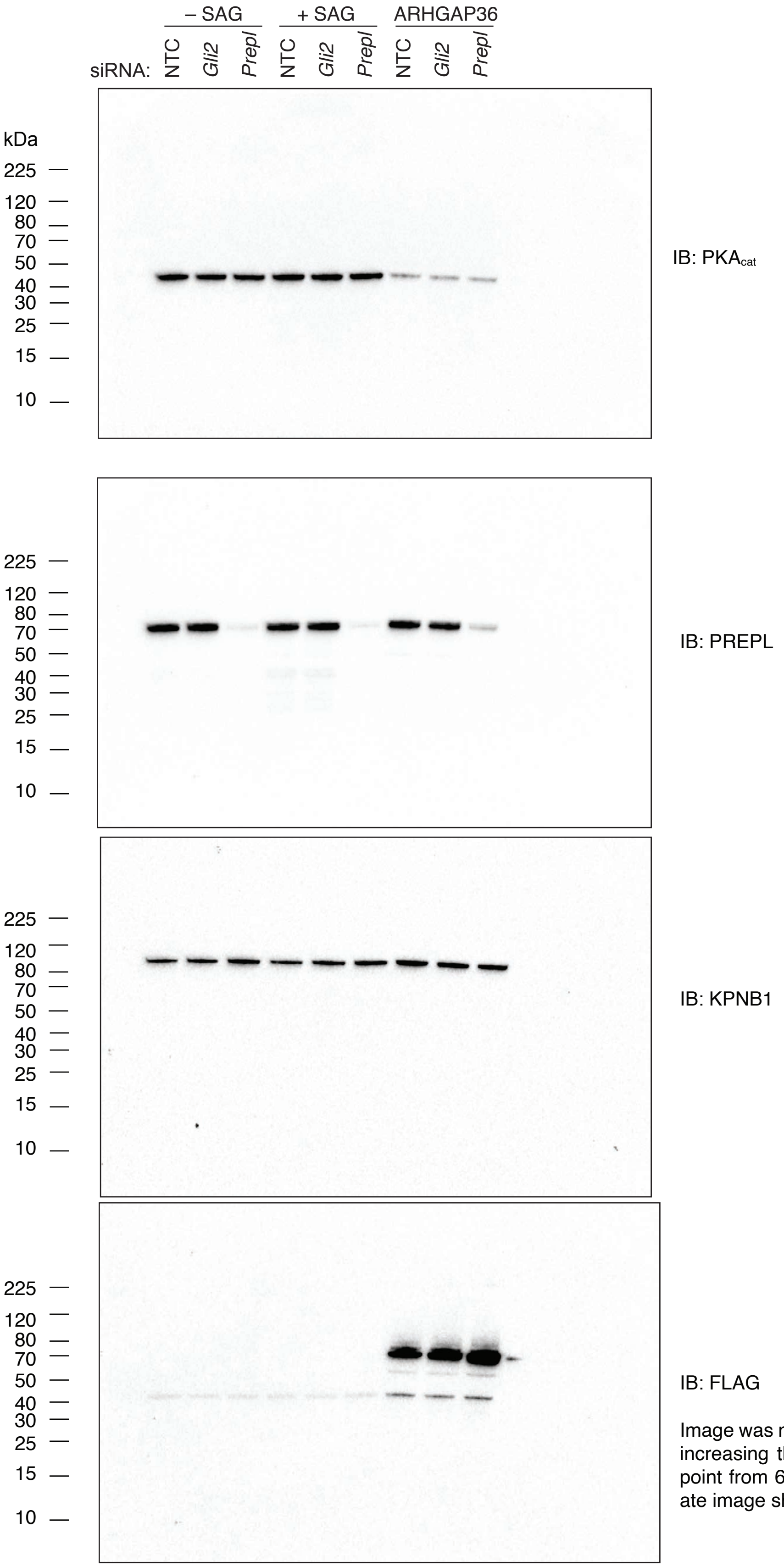

Image was modified in ImageLab by increasing the maximum grayscale point from 6284 to 37704 to generate image shown in Fig. 6C.

Figure 6B. Samples are NIH-3T3 cells stimulated with SAG or transduced with FLAG-tagged ARHGAP36 isoform 2 or 3 and transduced with indicated cDNA. Cells transduced with 3xFLAG-ires-mCherry vector were included as negative controls. Lanes not included in the figure are indicated by the red X's.

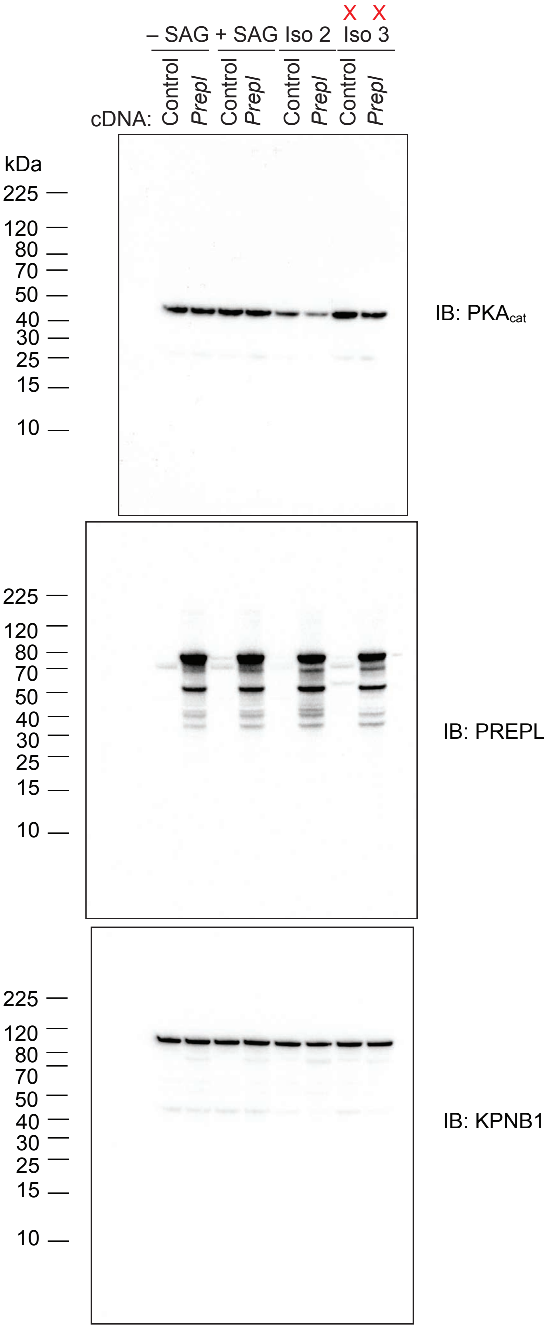

Figure 6D. Samples are NIH-3T3 cells stimulated with SAG or transduced with FLAG-tagged ARHGAP36 isoform 2 or 3 and transduced with indicated cDNA. Cells transduced with 3xFLAG-ires-mCherry vector were included as negative controls. Lanes not included in the figure are indicated by the red X's. Replicate 2 are samples from a different biological replicate that were included in quantification (Figure 6D) but not shown as a representative western blot.

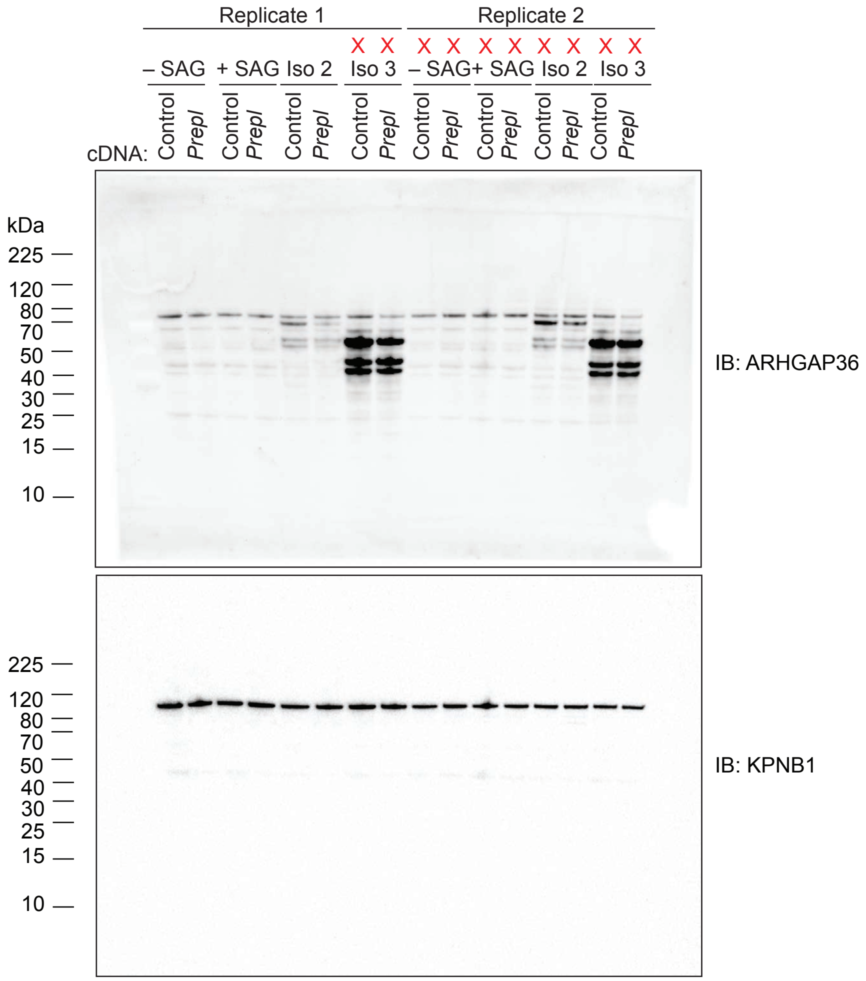

S5 Figure. Samples are the input and immunoprecipitates (IPs) of HEK-293 cells co-transfected with FLAG-tagged N3 and either GFP or the GAP-like domain fused to a LAP tag (S-tag-Precission protease site-GFP). Anti-FLAG IP was not as efficient as anti-GFP IP protocol and therefore not included in main paper. Both anti-FLAG and anti-GFP IPs used the same exact lysate as input. Lanes not included in the figure are indicated by the red X's.

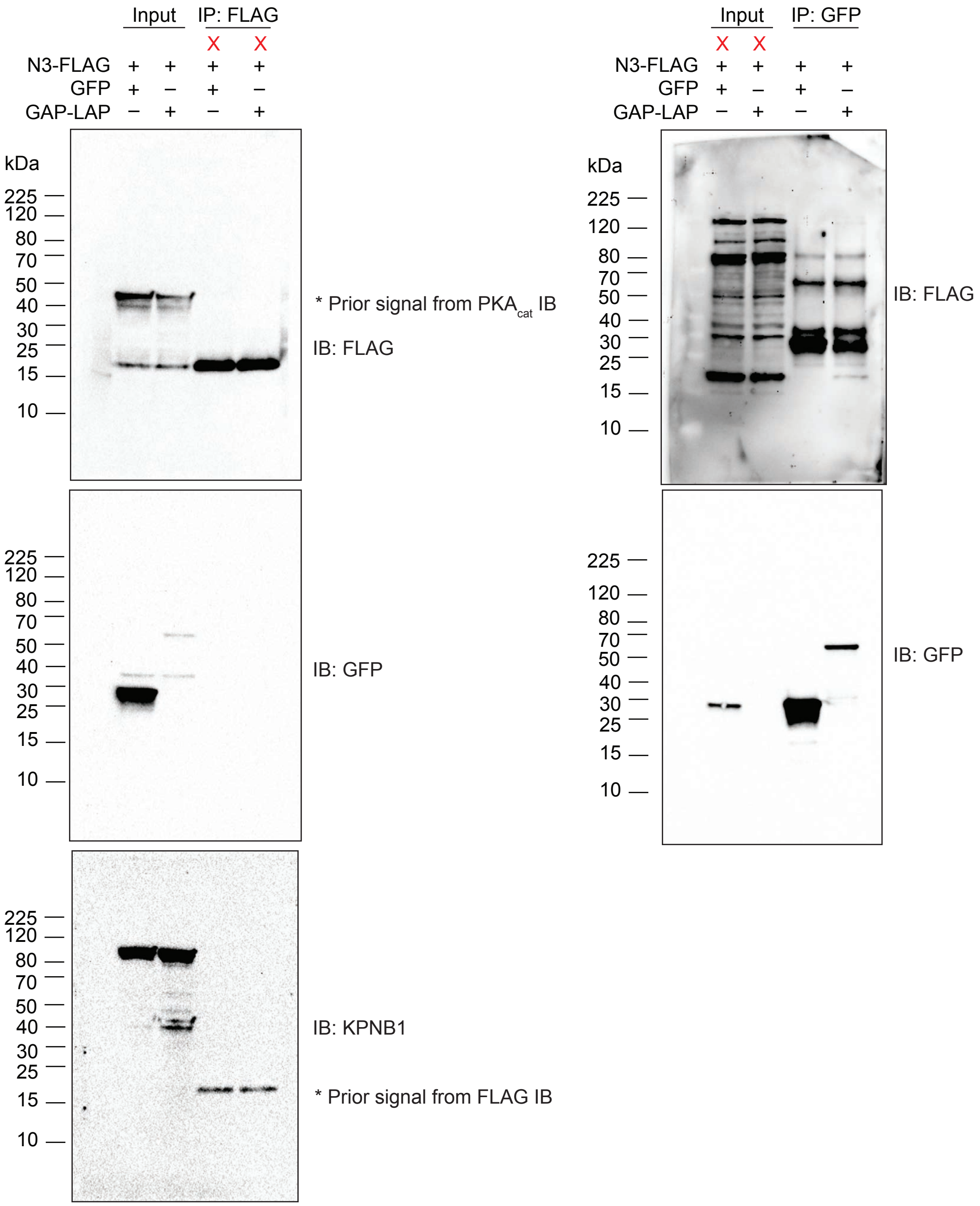

S6A Figure. Samples are SHH-LIGHT2 cells stimulated with SAG or retrovirally transduced with FLAG-tagged ARHGAP36 isoform 2 and transfected with indicated siRNAs. NTC indicates non-targeting control siRNA.

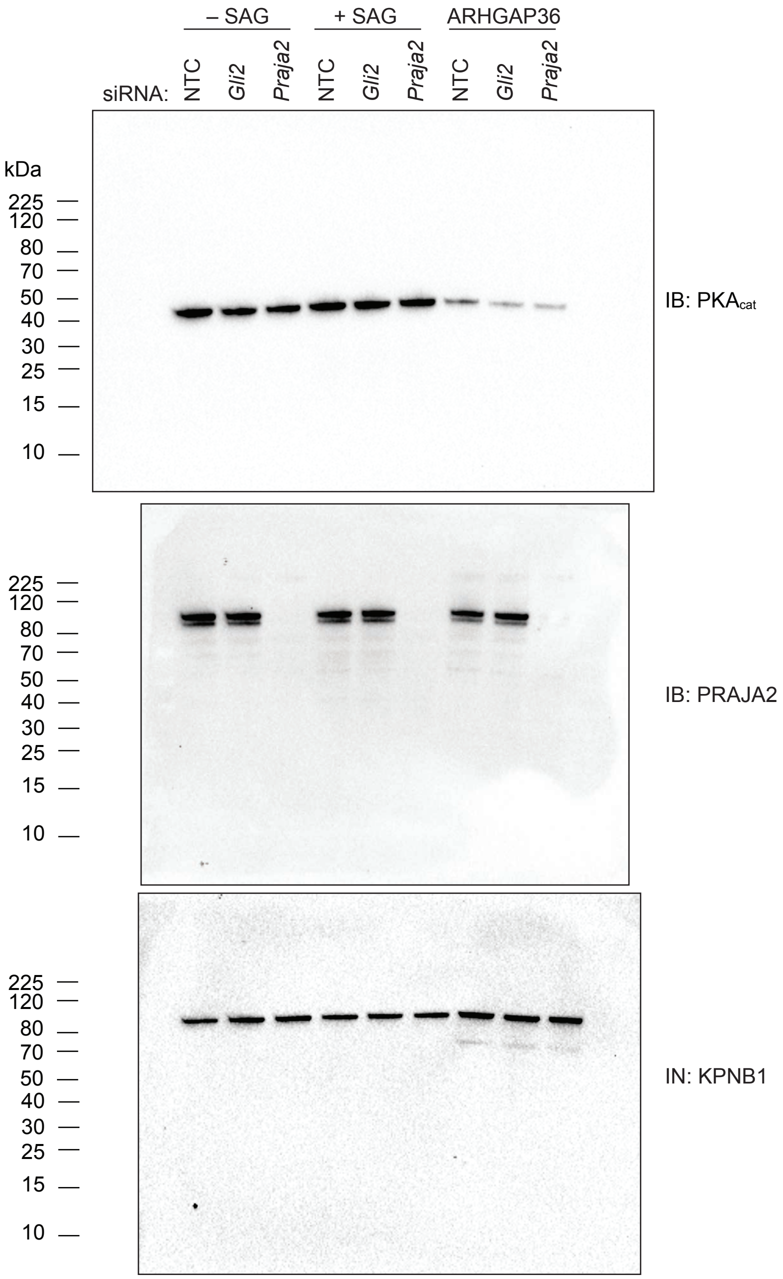

S6B Figure. Samples are NIH-3T3 cells stimulated with SAG or transduced with FLAG-tagged ARHGAP36 isoform 2 or 3 and transduced with indicated cDNA. Cells transduced with 3xFLAG-ires-mCherry vector were included as negative controls. Lanes not included in the figure are indicated by the red X's.

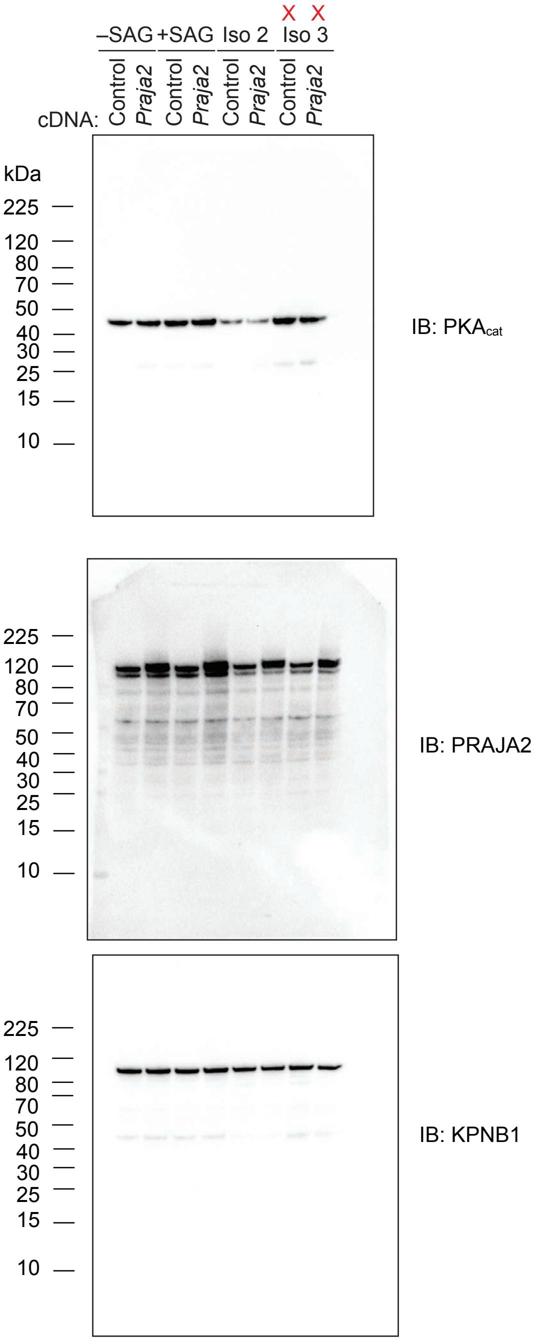

S7 Figure. Samples are the inputs and immunoprecipitates (IPs) of NIH-3T3 cells retrovirally transduced with the indicated FLAG-tagged ARHGAP36 truncation mutants.

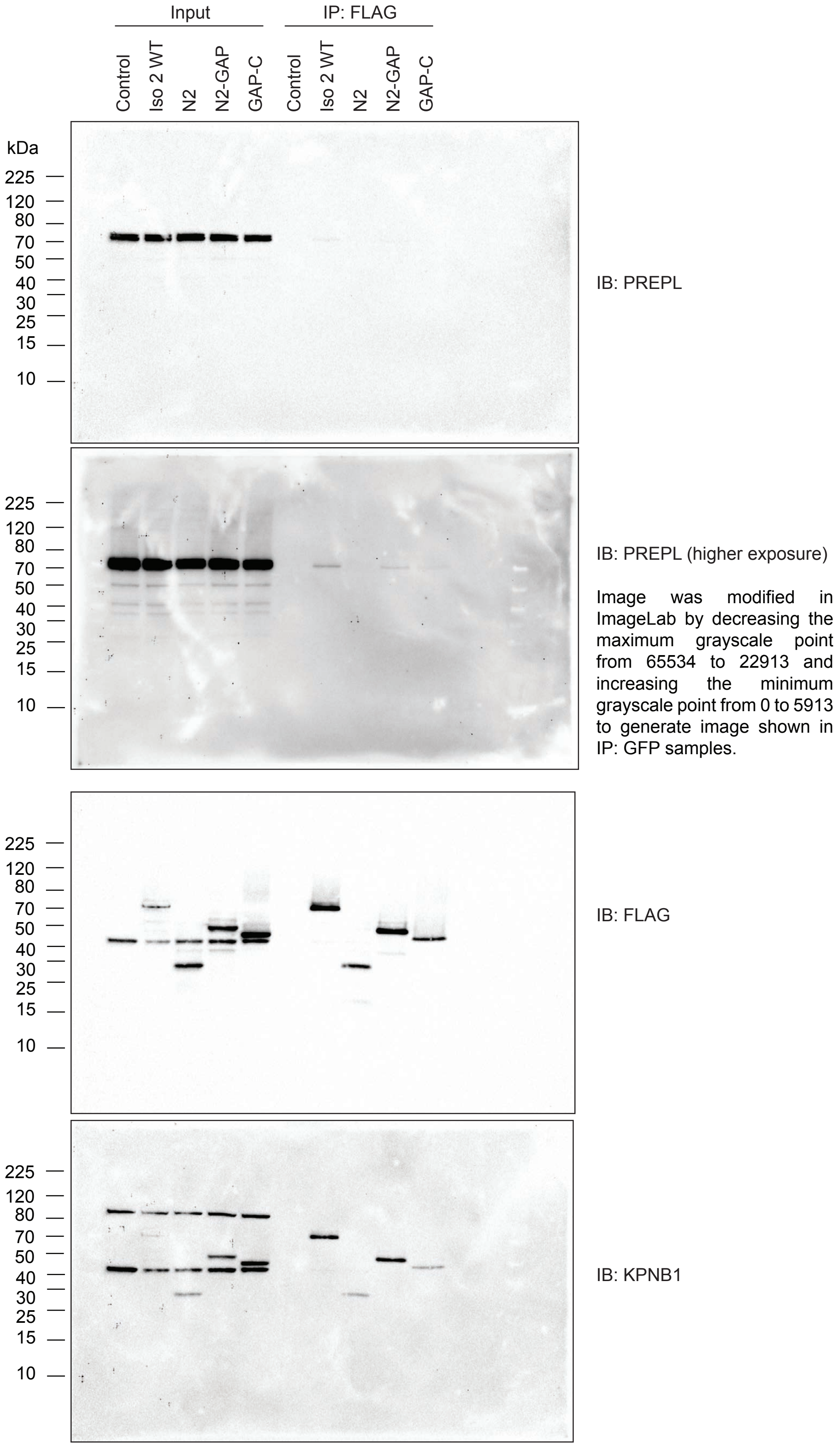

Supplement: S1 Raw images — (PDF) [file pone.0251684.s011.pdf]
