# Supplementary material for: Structure-activity mapping of ARHGAP36 reveals regulatory roles for its GAP homology and C-terminal domains
Source: PLoS One. 2021 May 17;16(5):e0251684. doi: 10.1371/journal.pone.0251684 (PMC8128262; doi:10.1371/journal.pone.0251684)
Supplement: S4 Fig — (PDF) [file pone.0251684.s004.pdf]

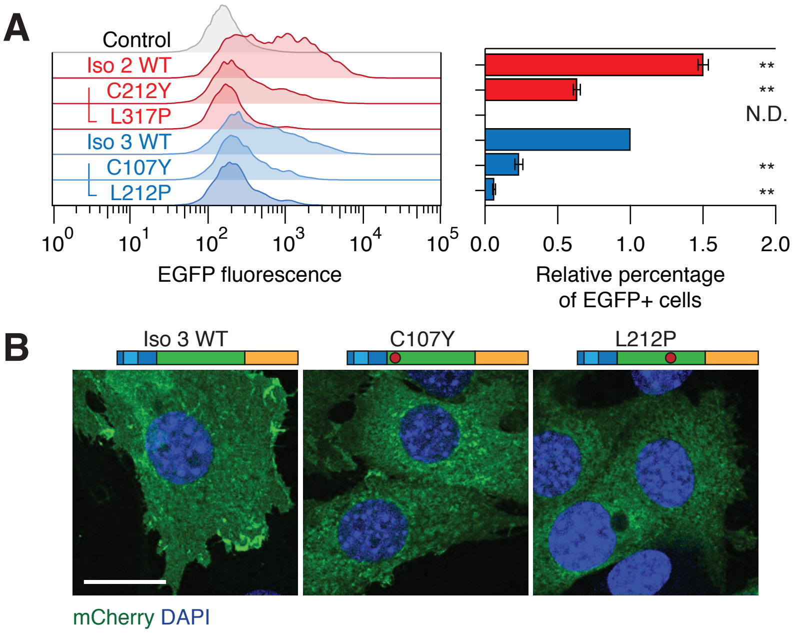

S4 Fig. Point mutations in the GAP-like domain attenuate the activity and localization of ARHGAP36 isoform 3. (A) Effects of GAP-like domain point mutations on isoform 3-mediated Gli activation, as assessed in the flow cytometry-based SHH-EGFP assay. mCherry fluorescence intensities were used to gate cells with comparable levels of ARHGAP36 expression, and the distributions of EGFP fluorescence (left) and relative percentage of EGFP+ cells (right) are shown for each ARHGAP36 construct. Data are the average fold change in percentage of EGFP+ cells relative to that of cells expressing wild-type ARHGAP36 isoform 3 for three biological replicates ± s.e.m. Double asterisks indicate $P < 0.01$ and N.D. indicates that no EGFP+ cells were detected in these populations. (B) Subcellular distributions of the indicated mCherry-tagged ARHGAP36 constructs in SHH-EGFP cells. Representative maximum-intensity Z-stack projections are shown with immunofluorescent staining for mCherry and DAPI (nucleus). Scale bar: 20 μm.
